# Genetic, Epigenetic and Phenotypic Diversity of Four *Bacillus velezensis* Strains Used for Plant Protection or as Probiotics

**DOI:** 10.3389/fmicb.2019.02610

**Published:** 2019-11-15

**Authors:** Oleg N. Reva, Dirk Z. H. Swanevelder, Liberata A. Mwita, Aneth David Mwakilili, Dillon Muzondiwa, Monique Joubert, Wai Yin Chan, Stefanie Lutz, Christian H. Ahrens, Lylia V. Avdeeva, Maksim A. Kharkhota, Donatha Tibuhwa, Sylvester Lyantagaye, Joachim Vater, Rainer Borriss, Johan Meijer

**Affiliations:** ^1^Centre for Bioinformatics and Computational Biology, Department of Biochemistry, Genetics and Microbiology, University of Pretoria, Pretoria, South Africa; ^2^Biotechnology Platform, Agricultural Research Council, Pretoria, South Africa; ^3^Department of Pharmaceutical Microbiology, Muhimbili University of Health and Allied Sciences, Dar es Salaam, Tanzania; ^4^Department of Molecular Biology and Biotechnology, University of Dar es Salaam, Dar es Salaam, Tanzania; ^5^Department of Plant Protection, Swedish University of Agricultural Sciences, Alnarp, Sweden; ^6^Department of Biochemistry, Genetics and Microbiology, University of Pretoria, Pretoria, South Africa; ^7^Forestry and Agricultural Biotechnology Institute, DST-NRF Centre of Excellence in Tree Health Biotechnology, University of Pretoria, Pretoria, South Africa; ^8^Agroscope, Molecular Diagnostics, Genomics and Bioinformatics and SIB Swiss Institute of Bioinformatics, Wädenswil, Switzerland; ^9^Department of Antibiotics, D.K. Zabolotny Institute of Microbiology and Virology, Kyiv, Ukraine; ^10^Robert Koch Institut, Berlin, Germany; ^11^Institut für Biologie, Humboldt Universität zu Berlin, Berlin, Germany; ^12^Department of Plant Biology, Linnéan Center for Plant Biology, Uppsala Biocenter, Swedish University of Agricultural Sciences, Uppsala, Sweden

**Keywords:** *Bacillus velezensis*, biocontrol, biopesticide, comparative genomics, epigenetics, gene regulation, genome sequencing, plant growth promoting rhizobacteria

## Abstract

*Bacillus velezensis* strains are applied as ecologically safe biopesticides, plant growth promoting rhizobacteria (PGPR), and in veterinary probiotics. They are abundant in various environments including soil, plants, marine habitats, the intestinal micro-flora, etc. The mechanisms underlying this adaptive plasticity and bioactivity are not well understood, nor is it clear why several strains outperform other same species isolates by their bioactivities. The main objective of this work was to demonstrate versatility of bioactivities and lifestyle strategies of the selected *B. velezensis* strains suitable to serve as model organisms in future studies. Here, we performed a comparative study of newly sequenced genomes of four *B. velezensis* isolates with distinct phenotypes and isolation origin, which were assessed by RNA sequencing under the effect of root exudate stimuli and profiled by epigenetic modifications of chromosomal DNA. Among the selected strains, UCMB5044 is an oligotrophic PGPR strain adapted to nutrient poor desert soils. UCMB5113 and At1 are endophytes that colonize plants and require nutrient rich media. In contrast, the probiotic strain, UCMB5007, is a copiotroph, which shows no propensity to colonize plants. PacBio and Illumina sequencing approaches were used to generate complete genome assemblies, tracing epigenetic modifications, and determine gene expression profiles. All sequence data was deposited at NCBI. The strains, UCMB5113 and At1, show 99% sequence identity and similar phenotypes despite being isolated from geographically distant regions. UCMB5007 and UCMB5044 represent another group of organisms with almost identical genomes but dissimilar phenotypes and plant colonization propensity. The two plant associated strains, UCMB5044 and UCMB5113, share 398 genes putatively associated with root colonization, which are activated by exposure to maize root exudates. In contrast, UCMB5007 did not respond to root exudate stimuli. It was hypothesized that alterations in the global methylation pattern and some other epigenetic modifications enable adaptation of strains to different habitats and therefore may be of importance in terms of the biotechnological applicability of these bacteria. Contrary, the ability to grow on root exudates as a sole source of nutrients or a strong antagonism against phytopathogens showed by the strains *in vitro* cannot be considered as good predictors of PGPR activities.

## Graphical Abstract

*Leptosphaeria maculans* suppression *in vivo* and protection of *A. thaliana* seedlings from this pathogen by *B. velezensis* strains.

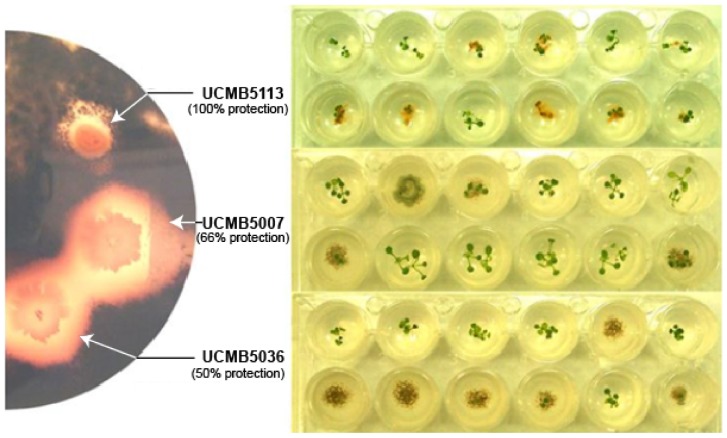

## Introduction

The application of the beneficial rhizobacterial *Bacillus velezensis* strains for plant disease biocontrol and plant growth promotion is a popular research topic with, on average, more than 100 papers annually introducing new plant growth promoting and protective *B. velezensis* isolates or applications. Indeed, the biosynthetic arsenal of this species seems inexhaustible with various antibiotics, enzymes, plant hormones and triggers of plant innate immunity responses being reported. Several recent reviews by Ye et al. (2018), Borriss et al. (2019), and Rabbee et al. (2019) provide a comprehensive overview of the inventory of bioactive metabolites of this species and its applications in agriculture. *B. velezensis* is the conspecific species introduced by integrating the formerly independent taxa *B. amyloliquefaciens* ssp. *plantarum*, *B. methylotrophicus* and ‘*B. oryzicola*’ (Dunlap et al., 2016). This organism devotes more than 8% of the total genetic capacity to synthesis of secondary metabolites to cope with competing microorganisms in the plant rhizosphere providing high rhizosphere competence (Chen et al., 2009a). A dozen non-ribosomal polypeptides with antibacterial, antifungal, nematicidal and/or regulatory functions are synthesized by different strains of this species. Amongst these, bacillaene, bacillibactin, bacillomycin, bacilysin, difficidin, fengycin and surfactin biosynthetic operons are integral parts of the majority of sequenced *B. velezensis* genomes (Koumoutsi et al., 2004; Schneider et al., 2007; Chen et al., 2009b, c; Liu et al., 2013; Wu et al., 2014, 2015; Gu et al., 2017; Lu et al., 2018; Kim et al., 2019). Furthermore, these bacteria can synthesize various low molecular weight metabolites and enzymes that mobilize inorganic nutrients from soil (Idriss et al., 2002), induce systemic resistance responses in plants to pathogens and improve abiotic stress management (Ryu et al., 2004; Li et al., 2015; Wu et al., 2018); and suppress growth of bacterial and fungal pathogens (Yuan et al., 2012; Raza et al., 2016). The ability to form biofilms on plant roots and colonize inner tissues of plants is also of significance for biocontrol activities of these bacteria (Krober et al., 2016; Al-Ali et al., 2018). Polysaccharides and teichoic acids produced by *B. velezensis* contribute to biofilm formation and increase drought tolerance in plants (Lu et al., 2018; Xu et al., 2019).

The applicability of *B. velezensis* strains as probiotics is less studied. However, it is generally believed that polypeptide antibiotics produced by the bacteria can inhibit intestinal pathogens. In addition, immune system modulation by these bacteria plays an important role (Sorokulova et al., 2008; Wang et al., 2009; Cutting, 2011; Horosheva et al., 2014; Elshaghabee et al., 2017; Ye et al., 2018).

Publications on the use of *B. velezensis* in agriculture as biocontrol agents or as probiotics mostly focus on cases of successful applications of selected strains in controlled environment or under field conditions. However, little or no information is presented to allow activity benchmarking of the reported strains compared to strains already used in bioproducts. Although huge amounts of literature data exist, it is still difficult to answer the question as to the extent different isolates of *B. velezensis* vary in terms of their biosynthetic capacities, biocontrol activities, and propensity to colonize plants or survive in animal intestines. An overview of the biological traits of microorganisms of the *Bacillus subtilis* group was recently published but did not address the strain or clonal level diversity (Alina et al., 2015).

Detailed studies on the general biology and versatility of *B. velezensis* were facilitated by the introduction of several model strains used worldwide for benchmarking. The strain, *B. velezensis* FZB42 (formerly *B. amyloliquefaciens* ssp. *plantarum* FZB42), was suggested as a paradigm for spore-forming PGPR microorganisms (Fan et al., 2018; Borriss et al., 2019). A complete genome sequence of this strain is available (Chen et al., 2007). More than 90 publications have reported studies using this strain as a model organism in different laboratories. These publications help to consolidate our knowledge about intrinsic mechanisms employed by this bacterium to colonize the plant rhizosphere and cope with bacterial and fungal competitors. However, information about the natural diversity of other bioactive *B. velezensis* strains remaines limited.

Another model organism, *B. velezensis* UCMB5113 (formerly *B. amyloliquefaciens* UCMB5113), has been introduced and used in several trials (Asari et al., 2016, 2017a,b; Abd El-Daim et al., 2018). This strain was originally isolated from soil in Zakarpatye (Uzhgorod, Ukraine) by Dr. Boris M. Sharga (Uzhgorod National University) and deposited into the Ukrainian Collection of Microorganisms (UCMB) in the Institute of Microbiology and Virology, Kyiv (Ukraine). In contrast to the predominantly non-colored colony phenotype of *B. velezensis* strains, UCMB5113 produces an orange pigmentation during all growth stages in both solid and liquid media (Reva et al., 2004). This strain outperforms other *B. velezensis* in its ability to colonize plants, which was confirmed *in vitro* on oilseed rape (*Brassica napus*) using qPCR (Johansson et al., 2014). After treatment of oilseed rape seeds with this strain, peculiar orange colonies were isolated even from the second-generation seeds after surface sterilization (unpublished results). These observations suggest that this strain is intimately related to plants. Complete genome sequencing confirmed the strain affiliation with *B. velezensis*, but with several substantial differences in gene numbers and the genomic organization when compared to the type strain *B. velezensis* FZB42 (Niazi et al., 2014).

Other studies showed that natural *B. velezensis* isolates differ in their cultivation preferences and their ability to colonize plant root, promote plant growth or protect plants from phytopathogens. Here, two *B. velezensis* strains, UCMB5007 and UCMB5044, that are genetically distant from both FZB42 and UCMB5113, were selected to undergo comparative studies with the model plant growth promoting strain *B. velezensis* UCMB5113. The strain At1 isolated at Swedish University of Agricultural Sciences (Uppsala, Sweden) from seedlings of *Arabidopsis thaliana* germinated aseptically from surface sterilized seeds (Reva et al., 2004) is an example of another *B. velezensis* strain producing colonies with orange pigmentation like UCMB5113. This strain was included to investigate the genetic diversity of *B. velezensis* endophytes producing orange pigmentation which were isolated from geographically distant regions.

UCMB5007 was isolated from calf gut on a farm in the Kyiv region (Ukraine) and used as the stock-raising probiotic Bacterin-SL^TM^. The strain was considered to be a part of the transient micro-flora of calf intestines. *In vitro*, this strain inhibits a wide range of bacterial and fungal test-cultures including both intestinal pathogens and phytopathogens. However, its ability to control pathogens *in planta* and promote plant growth was rather limited, probably due to its disinclination in plant colonization. UCMB5044 was isolated from surface sterilized cotton stems (*Gossypium* sp.) cultivated under field conditions in Tajikistan (Reva et al., 2004). UCMB5044 shows oligotrophic growth preferences, growing better on minimal rather than energy rich media. In contrast, UCMB5007, UCMB5113 and At1 are copiotrophs requiring nutrient-rich media for growth. A detailed overview on oligotrophic and copiotrophic bacterial lifestyle was published by Koch (2001). The current work aimed at a comparative study of complete genome sequences, gene regulation patterns and epigenetic modifications in chromosomal DNA of these four selected strains to evaluate mechanisms involved in adaptive evolution of *B. velezensis*.

## Materials and Methods

### Maize and Oilseed Rape Root Exudate Preparation

Seeds of the maize cultivar F021SW kindly provided by Mr. Lebogang Madubanya (Agricultural Research Council Maize Breeding Section, Potchefstroom, South Africa) and *Brassica napus* seeds (Larissa spring variety line, Scandinavian Seed AB made available by the Swedish University of Agricultural Sciences, Uppsala) were surface sterilized by soaking in 70% ethanol for 5 min, followed by shaking in 15% bleach solution supplemented with 10 μl Tween 20 (Merck) for 15 min, and then triple washed by sterile, double distilled water and left in 50 ml tubes with water overnight.

Maize seeds were placed on four wet filter paper sheets and incubated in the dark at 28°C for 4 days to allow germination. Seedlings with roots around 1 cm were moved to 15 ml falcon tubes with sterile tap water and hold above the water level using sterile P1000 pipette tips. Tubes were covered below the seed with foil to stimulate root growth and incubated for a week in a growth chamber with controlled 18/6 h light/dark photoperiod and 25/20°C day/night regime. After the incubation period, seeds were evaluated for possible contamination. The liquid with root exudates was collected, pooled to provide a consistent treatment for all the lines, sterilized by filtering (0.22 μm pores) and kept frozen in 50 ml aliquots. The dry mass of root exudates detected by lyophilization was 20 mg per 100 ml (0.02%).

Oilseed rape seeds were pre-germinated in Petri dishes containing 0.2x Murashige-Skoog medium (Duchefa Biochemie B.V., Haarlem, Netherlands) mixed with 0.5% plant agar. Seedlings of the same size were selected and transferred to a 250 ml flask containing 100 ml of 0.5x Murashige-Skoog medium. Seedlings were grown for 2 weeks at 22°C under 16/8 h light/dark photoperiod with shaking (Infors AG, Basel, Switzerland) at 110 rpm. Root exudates were collected after 2 weeks, controlled for contamination, pooled and lyophilized. The dry residues of root exudates were dissolved to the required concentration, sterilized by filtering (0.22 μm pores) and kept in 50 ml aliquots in the cold room. The root exudates working solutions of 1% and 10% were prepared from the stock solution.

### Cultures, Growth Curves and Growth Conditions

*Bacillus velezensis* strains UCMB5007, UCMB5044 and UCMB5113 were obtained from the Ukrainian Collection of Microorganisms, section Bacteria (UCMB) at the D.K. Zabolotny Institute of Microbiology and Virology, Kyiv (Ukraine). The strain *B. velezensis* At1 is available at the University of Pretoria (South Africa). Lyophilized stock cultures were kept at room temperature or as frozen spore suspensions at −20°C. The strains were cultured in Luria broth (LB, Duchefa Biochemie) liquid or solid medium at 28°C or 37°C; and in M9 minimal salt medium (Sigma-Aldrich Co, St Louis, MO, United States) containing mineral salts and 0.4% glucose, or with root exudates instead of glucose. *Bacillus* spores were harvested after 5 days of cultivation on LB agar plates at 37°C and re-suspended in sterile water to achieve 10 OD at 600 nm.

Growth curves of bacteria were determined in LB broth, M9 and M9 media supplemented with 1 or 10% oilseed rape (*Brassica napus* cv. Larissa) root exudates incubated in 96-well plates on a plate reader spectrophotometer (FLUOstar Omega, BMG LABTECH) at 28°C for 16 h and 22 min, with shaking. OD measurements were recorded automatically at 8 min intervals. Four cell replicates, including negative controls with the selected media and water added instead of bacterial suspensions, were performed for all growth conditions. The negative control cells’ OD values were subtracted from cells with bacterial growth. OD records for every 30 min were combined and an average OD was calculated.

### Antagonistic Activity Assays

The antagonistic activity of *B. velezensis* strains was determined *in vitro*, with potato dextrose agar (PDA, Sigma-Aldrich Co) against phytopathogens and with LB agar against entero-pathogens. *Bacillus* cultures were inoculated onto the center of plates with a diameter of 5 mm using a microbiological loop and the plates were incubated for 3 days at 28°C. Bacterial test-cultures obtained from UCMB were inoculated making a streak on the medium surface with the microbiological loop from the edge of the plate toward the *Bacillus* colony leaving a clearance zone 2 mm wide. The plates were cultivated further for 24 h at 28°C (phytopathogens) or 37°C (entero-pathogens). Zones of inhibition were recorded in millimeters.

Fungal phytopathogens were obtained from the culture collection at the University of Dar es Salaam (Tanzania). The fungal strains were grown on PDA plates at 28°C until spore production. Spores were harvested by adding sterile water to the plates and scraping the agar surface with a flamed spatula. Spore suspensions were adjusted with sterile water to the standard OD of 10 OD at 600 nm. Aliquots of 200 μl of spore suspensions were added to 400 ml melted and cooled sterile LB agar and gently mixed prior to pouring into sterile plates. After solidifying, 5 mm sterile filter paper disks were placed in the center of the plate using flamed forceps. Aliquots of 10 μl (1 OD at 600 nm) *Bacillus* spore suspension were pipetted onto each of the disks. Plates were sealed and incubated at 28°C for 48 h. Zones of fungal growth inhibition were measured from the edge of the 5 mm disk to the end of the inhibition zone. All bacterial and fungal antagonistic assays were performed in triplicate and average values calculated.

### *Ralstonia solanacearum* Biocontrol Assays on Tomato Seedlings

A *Ralstonia solanacearum* isolate causing bacterial wilt of tomato was obtained from the culture collection of phytopathogens at the University of Dar es Salaam (Tanzania). The isolate was cultured on LB plates at 37°C for 24 h. A stock suspension of 10 OD (at 600 nm) was obtained by flooding each plate with sterile water prior to scraping the bacteria off using a flamed spatula.

Tomato seeds were surface sterilized by soaking them in 0.1% sodium hypochlorite for 3 min and then rinsed four times with sterile water. Individual seeds were treated separately by soaking each for 6 h in a *Bacillus* spore suspension (1 OD at 600 nm) prepared as described above. Control seeds were soaked with sterile water for the same time. Seeds were air dried after treatments before being planted into sterilized soil in the nursery, each kept separate according to the treatment. Pots were watered every other day for 4 weeks. The seedlings were then transferred onto sterile soil in pots, three seedlings per pot, in five replicates for every treatment. Seedlings were then transplanted as three seedlings per pot into sterilized soil, with five pot replicates for each treatment. After 7 days, 200 ml *R. solanacearum* working suspension was applied as a drench onto each of the *Bacillus* treated and positive control pots. Negative control pots were uninfected. Seedlings were watered every other day during the experiment. Symptom development and disease progression were recorded every 7 days for a month post pathogen treatment. Tomato wilting was scored according to the following disease index: 0 – no symptoms (healthy plant); 1 – one leaf partially wilted; 2 – two to three wilted leaves; 3 – all but one to three leaves wilted; 4 – all leaves wilted; 5 – plant dead. Disease progression curves were plotted and the AUDPC were calculated using the recorded indices. A smaller AUDCP indicates better disease prevention.

### Pigment Synthesis and Extraction

Pigment production were investigated by culturing the bacterial strains in a liquid synthetic medium consisting of sodium citrate (1.29 g/l), (NH_4_)_2_HPO_4_ (4.75 g/l), KH_2_PO_4_ (9.6 g/l), MgSO_4_ (0.18 g/l), and glucose (10 g/l). The medium was solidified with 1% agar (Merk). Plates were inoculated with 10^6^–10^7^ CFU/ml bacterial inoculums and cultivated for 24 h at 37°C. Pulcherrimin was extracted from cells with 1:1 methanol (100%) and KOH-water (50:50) solution (MacDonald, 1965). *Bacillus subtilis* UCMB5114 actively produce pulcherrimin and was used as the positive control.

### MALDI-TOF MS and HPLC ESI MS Detection of Lipopeptides, Siderophores and Polyketides

*Bacillus velezensis* strains were grown in Landy liquid medium (Landy et al., 1948), where the supernatants were collected at 24 and 48 h for secondary metabolite study. Lipopeptides, siderophores and polyketides were identified by matrix-assisted laser desorption ionization-time of flight (MALDI-TOF) Mass Spectrometry, where the filtered supernatants were extracted with 50% acetonitrile/0.1% trifluoroacetic acid. The matrix solution of 2,5-dihydroxbenzoic acid was used. Two μl portions of extracts and 2 μl of matrix solution were mixed, spotted onto the target, and air dried. The MALDI-TOF MS were measured as described by Vater et al. (2002). Bruker Autoflex MALDI-TOF instrument containing a 337-nm nitrogen laser was used to capture the mass spectra reading of desorption and ionization.

High-performance liquid chromatography (HPLC) separation of the bioactive compounds produced by the investigated *B. velezensis* strains was performed on an Agilent 1200 HPLC system (Agilent, Waldbronn, Germany). Aliquots of acetonitrile (ACN)-water extracts of the freeze-dried supernatants were fractionated by reversed-phase HPLC on a Zorbax Eclipse XDB HPLC C18 3,5-μm (4,6 by 150 mm) column at a flow rate 1.5 ml/min with an increasing gradient from 0% ACN to 100% ACN (with 0.1% formic acid) in 15 min. In HPLC chromatograms (Supplementary Figures 1–5), the eluted compounds were recorded by measurement of the OD values at two wavelengths, 365 and 230 nm, then compared to the standard values characteristic for the non-ribosomal peptide synthetases (NRPS) products as published before (Chen et al., 2006).

### DNA Extraction and Sequencing

DNA was extracted from overnight liquid LB cultures grown at 37°C with the GenElute Bacterial Genomic DNA Kit (Sigma-Aldrich, Johannesburg, South Africa). The UCMB5007 and UCMB5044 genomes were sequenced using paired-end library preparation and an Illumina HiSeq 2000 at Macrogen Inc. (Republic of Korea). SMRT sequencing was carried out on a PacBio RSII machine (1 SMRT cell per strain) at the FGCZ, Zurich, Switzerland. Size selection was performed using the BluePippin system (SAGE Science, United States) as described before (Omasits et al., 2017) and resulted in fragments with an average subread length of 8–10 kb. Two 2 × 300 bp Illumina paired end libraries were prepared per strain using the Nextera XT DNA kit and sequenced on a MiSeq at Agroscope (Wädenswil, Switzerland). The At1 genome was sequenced with PacBio RSII CCS technology at Inqaba Biotec Ltd. (Pretoria, South Africa). DNA quality was evaluated spectrophotometrically at 260 and 280 nm light absorbance with a NanoDrop 1000 spectrometer (Thermofisher, South Africa). All raw DNA and RNA reads generated for this project by Illumina and PacBio sequencers are available from NCBI SRA database records accessible through the BioProject Web-sites. BioProject accession numbers are indicated in Table 1.

**TABLE 1:** NCBI accession numbers of WGS sequences and metadata of *B. velezensis* strains used in this study.

**Strain**	**BioSample**	**BioProject**	**WGS**
UCMB5007	SAMN12015780	PRJNA176687	CP041143.1
UCMB5044	SAMN12015793	PRJNA548267	CP041144.1
UCMB5113	SAMEA2272338	PRJEB1418	HG328254.1
At1	SAMN12058370	PRJNA176703	CP041145.1

### Genome Assembly and Annotation

PacBio RSII subreads were extracted using the SMRT Portal and protocol RS_HGAP_Assembly.3 (default parameters with minimum subread length set for 1000, estimated genome size of 4 Mb). The subreads were then *de novo* assembled using Flye v.2.3.3 (Kolmogorov et al., 2019), similar as described before (Schmid et al., 2018). Default parameters were applied except for the estimated genome size set to 4 Mb. The assemblies of strains UCMB5007 and UCMB5044 resulted in one contig each, which were start-aligned by the gene *dnaA* and polished using two Quiver runs and the RS_Resequencing.1 protocol on the SMRT Portal with parameters set by default. For the strain UCMB5007, one region of ∼30 kb consisting of multiple rRNA operons could not be fully resolved. The assemblies were then further polished using 2 × 300 bp Illumina reads and 1–2 Freebayes v.1.2.0 runs with minimum alternate fraction: 0.5, minimum alternate count: 5 (Garrison and Marth, 2012) to correct any potentially remaining small sequencing errors. The filtered PacBio subreads were mapped to the polished and start-aligned contigs using *graphmap* v.0.5.2 (Sović et al., 2016) to verify the circularity and completeness of the assemblies. Variants were manually inspected in the Integrated Genome Viewer (Thorvaldsdóttir et al., 2013) and subsequently corrected using *bcftools* v.0.1.19 (Narasimhan et al., 2016). PlasmidSpades was run on the Illumina data as all PacBio approaches involved a BluePippin size selection step, which might have led to the removal of smaller plasmids.

The completeness of the final assemblies was evaluated using the benchmarking universal single-copy orthologous (BUSCO) software (Simão et al., 2015). Assembled genomes were annotated using the online RAST Server (Aziz et al., 2008)^1^ and manually curated in Artemis 14.0.0^2^ using the annotation of orthologous genes from the previously published UCMB5113 genome (Niazi et al., 2014). Gene orthologies were predicted by OrthoFinder (Emms and Kelly, 2015). Clusters of genes encoding biosynthesis of secondary metabolites were identified with antiSMASH (Blin et al., 2019). Adenylation and condensation domains in non-ribosomal polypeptide synthetases (NRPS), and their amino acid specificity, were identified with antiSMASH and the PKS/NRPS Analysis Web-tool^3^. Involvement of genes in metabolic pathways were modeled and analyzed with Pathway Tools 15.5 for 64-bit Windows (Karp et al., 2016), SubtiWiki and AmyloWiki Web resources (Zhu and Stülke, 2017; Fan et al., 2019). SeqWord Genome Island Sniffer (Bezuidt et al., 2009) was used to identify possible insertions of horizontally transferred genomic islands and the replication origin and terminus of the bacterial chromosomes using GC-skew between the leading and lagging strands (Freeman et al., 1998). The complete genome sequences and metadata of the *B. velezensis* strains in this work are available from NCBI (Table 1).

### RNA Extraction and Sequencing

Bacteria were grown overnight in LB broth at 28°C, with 200 rpm orbital shaking. Cells were pelleted by centrifugation (5 min at 5,000 rpm) and washed twice with sterile water. Collected bacterial cells were weighed and re-suspended in sterile water to achieve a 50 mg/ml concentration.

The resulting cell suspension (1 ml) was added to 4 ml of the maize root exudate (0.02%) solution or water (negative control). All tubes were incubated for 20 min at 28°C and then inactivated by the killing buffer, i.e., 20 mM Tris–HCl, 5 mM MgCl_2_, 20 mM NaN_3_ and pH 7.5 (Völker et al., 1994). Cells were pelleted with centrifugation (5 min at 5,000 rpm) at 4°C and the supernatant removed. Cell pellets were frozen and stored at −80°C until RNA extraction with the RNeasy Mini Kit (QIAGEN, Inqaba Biotech). Quality and concentration of extracted RNA samples were evaluated using Qubit fluorometric system and reagents (Thermo Fisher Scientific, South Africa). Fifty Units of RNase inhibitor provided with QIAGEN Kit were added to every 50 μl RNA sample. Samples were subsequently used in the RNAtag-Seq protocol (Shishkin et al., 2015) to prepare them for paired-end (2 × 125 bp) Illumina sequencing on a HiSeq 2500 instrument at the Agricultural Research Council’s Biotechnology Platform (Pretoria, South Africa). Root exudate exposure experiments were repeated five times and the negative control three times with every strain to allow statistical validation of the differential gene expression.

### Transcriptional Profiling

Reads obtained were demultiplexed with FASTQCMCF prior to being trimmed on quality (Phred score = 21) using the Raw RNA-Seq Data Processing pipeline implemented in UGENE 1.32.0 (Golosova et al., 2014). UGENE package was used to convert genome annotation in GenBank format to the GFF3 format required for transcriptional profiling. RNA subread sequence alignment against the reference sequence was performed using the R package *Rsubread* (Liao et al., 2019). Numbers of subreads overlapping predicted protein coding sequences were estimated by the function *featureCounts* (Rsubread package) using BAM alignment files and the GFF3 annotation files generated in the previous steps. Subsequently, the output files with feature counts were assigned to the control or experiment sets, which represent the repeats of RNA samples extracted either from bacterial suspensions in water (control condition), or in root exudates, for all the different strains grown under the same conditions. Normalization of reads and the statistical analyses of differential gene expression levels were performed by the Bioconductor DESeq2 R package (Love et al., 2014). Text output files of DESeq2 containing normalized read counts, fold change values and statistical validation parameters were visualized in scatter plots using in-house Python 2.7 scripts. The RNA subreads were also used to predict operon structures in sequenced genomes. Subread mapping was performed by the Bowtie2 algorithm implemented in UGENE 1.32.0.

### Profiling of Epigenetic Modifications

Tools available in the SMRT Link 6.0.0 software^4^ were used with an in-house Python script to generate a pipeline to do base call kinetic analysis on the PacBio reads generated from chromosomal DNA. The Python pipeline consists of the following seven steps. (i) The complete genome consensus sequences in FASTA format, obtained by assembling PacBio reads, were indexed by the program *samtools* (part of the SMRT Link package) to be used as the reference sequence for PacBio read alignment. (ii) PacBio reads were converted from the original BAX.H5 format to BAM format by the tool *bax2bam*. (iii) Reads stored in BAM files were aligned against the indexed reference sequence by the tool *blasr*. (iv) Aligned reads in BAM format were sorted by locations and indexed by *samtools sort* and *index* functions. Sorted BAM files of PacBio read alignments were deposited at the NCBI SRA database under corresponding BioProject accessions (Table 1). (v) Sorted and indexed BAM files were analyzed by the tool *ipdSummary* to evaluate the base call kinetics for every nucleotide in the reference genome (output file *^∗^_kinetics.csv*). Also, for every nucleotide position with a significant base call delay in multiple overlapped reads, the program calculated several statistical parameters such as the interpulse duration (IPD) ratio of the average base call time to the expectation, and the quality values (QV) scores. A QV score of 14 corresponds to p-value 0.05, and a QV score of 21 to *p*-value 0.01. The program stores all the estimated parameters together with context sequences into an output file *^∗^_basemods.gff*. (vi) Contextual motifs of base modifications were searched by the tool *motifMaker*. (vii) Epigenetic profiles of the studied genomes were visualized using an in-house Python script, which uses the *^∗^_kinetics.csv* and *^∗^_basemods.gff* output files.

The program *motifMaker* identifies nucleotide motifs associated with epigenetic modifications and differentiates between methylated sites and modifications of an unknown nature. Adenine residues methylated at 6th nitrogen atom are denoted m6A; with methylated cytosine residues denoted as m4C. Cytosine residues may, however, be methylated at either 4th or 5th carbon atom, but these are not distinguished as such by the program. While m4C notation is used throughout the text of the paper to denote methylated cytosine residues, it should be noted that this includes m5C methylated sites as well.

### Whole Genome Phylogenetic Inference

Phylogenetic relations between newly sequenced *B. velezensis* and the reference strains with available whole genome sequences (NCBI) were inferred by the supermatrix approach (Delsuc et al., 2005). In total, 16 complete genome sequences including the type cultures *B. velezensis* FZB42, *B. amyloliquefaciens* DSM7 and *B. subtilis* 168, were used in the analysis. OrthoFinder software (Emms and Kelly, 2015) identified 592 orthologous genes, excluding paralogs, in the selected genomes. Amino acid sequences encoded by these genes were aligned within each cluster of orthologous proteins prior to concatenating alignments into an artificial super-alignment, 223,182 amino acids in length. The phylogenetic tree was inferred using the Neighbor-Joining algorithm implemented in MEGA6 (Tamura et al., 2013).

## Results

### Strain Specific Growth Curves

**FIGURE 1:**
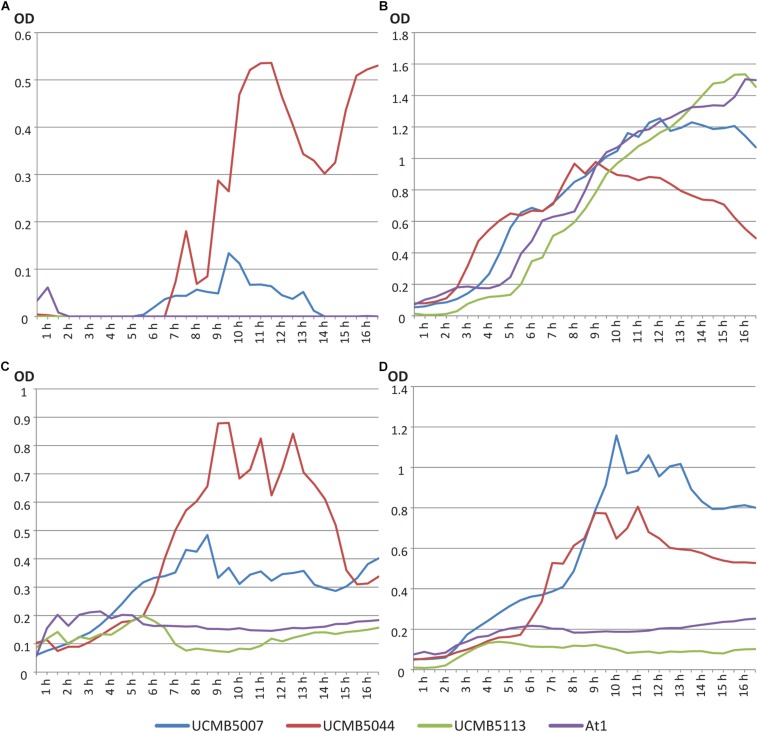
Growth curves of *B. velezensis* strains UCMB5007, UCMB5044, UCMB5113 and At1 on **(A)** LB broth; **(B)** M9 minimal medium with 0.4% glucose; **(C)** M9 supplemented with 1% oilseed rape root exudate; **(D)** M9 supplemented with 10% oilseed root exudate. Every point in the curves corresponds to an average value of 20 OD measurements – five records taken every 8 min in 4 repeats.

Growth curves of four *Bacillus velezensis* strains were evaluated in LB broth, M9 minimal medium with 0.4% glucose and M9 medium with 1% and 10% oilseed rape root exudates supplemented instead of glucose. OD measurements were taken automatically every 8 min. Data points of the growth curves shown in Figure 1 were calculated as average values of five continued measurements in four repeats per strain per medium.

UCMB5044 was the only strain able to grow on M9 minimal medium supplemented with glucose. On nutrient rich LB, this strain reached the stationary phase soon after the beginning of cultivation at lower cell density compared to the other strains. The growth curve of this strain on LB started to decline after 9 h in cultivation. Preferable growth on nutrient poor media is characteristic of oligotrophs. In contrast, UCMB5007 grew poorly on minimal M9 medium with 0.4% glucose but recovered growth in the presence of at least 1% root exudates. While UCMB5044 did not grow better when the root exudate levels were increased, the growth of UCMB5007 increased several-fold indicating strain specific rate-limiting compounds in the root exudates. For the strains UCMB5113 and At1, even 10% root exudate supplement did not support any active growth. According to known composition of root exudates (Lapie et al., 2019), 10% concentration corresponds approximately to 1% glucose or 1.8% glucose with other carbohydrates (fructose, mannose, cellobiose, xylose, mannitol and rhamnose). Also, root exudates provide organic acids, amino acids, vitamins and other various organic and inorganic compounds absent in the minimal medium.

UCMB5007, UCMB5113 and At1 grew vigorously on LB. Therefore, UCMB5113 and At1 can be defined as extreme copiotrophs requiring an environment rich in nutrients, with UCMB5007 showing an intermediate state between the oligotrophic and copiotrophic lifestyles. Observed fluctuations of the growth curves may be explained by self-regulated phase changes in the bacterial populations, i.e., between clumped growth in micro-colonies and flagellar swimming.

### Antagonistic Activity Against Pathogenic Bacterial and Fungal Strains *in vitro* and *in vivo*

The ability of three selected *B. velezensis* strains (excluding At1) to suppress the growth of various bacterial (Table 2) and fungal (Table 3) pathogenic test-cultures was evaluated in series of *in vitro* experiments. Bacterial test-cultures included both phytopathogens and entero-pathogens.

**TABLE 2:** Growth inhibition of different pathogenic bacteria by the three *B. velezensis* strains over 3 repeats.

**Bacterial pathogenic test-cultures**	**Zone of inhibition (mm), *n* = 3**		
	**UCMB5007**	**UCMB5044**	**UCMB5113**
*Agrobacterium tumefaciens*	17 ± 3	0	<2
*Bacillus cereus*	13 ± 2	8 ± 2	12 ± 2
*Clavibacter michiganensis*	0	<2	0
*Escherichia coli*	22 ± 3	0	17 ± 2
*Pectobacterium carotovorum*	9 ± 2	0	8 ± 1
*Klebsiella pneumonia*	15 ± 2	0	<2
*Micrococcus luteus*	30 ± 4	16 ± 3	17 ± 2
*Proteus vulgaris*	25 ± 3	9 ± 3	9 ± 3
*Pseudomonas aeruginosa*	0	0	0
*Ralstonia solanacearum*	17 ± 2	0	7 ± 2
*Salmonella enterica* var. *abortusequi*	16 ± 3	0	12 ± 1
*Salmonella typhimurium*	20 ± 2	0	13 ± 3
*Shigella sonnei*	20 ± 3	0	15 ± 2
*Serratia marcescens*	20 ± 3	0	12 ± 3
*Staphylococcus aureus*	25 ± 2	0	25 ± 3
*Xanthomonas campestris*	25 ± 3	0	11 ± 2
*Xanthomonas citris* var. *malvacearum*	12 ± 2	0	<2
*Stenotrophomonas maltophilia*	15 ± 3	0	13 ± 2

**TABLE 3:** Inhibition of growth of fungal phytopathogens by culture medium supernatant and spore suspensions of *B. velezensis* strains.

**Fungal phyto-pathogens**	**UCMB5007**		**UCMB5044**		**UCMB5113**	
	**Super-natant**	**Spore suspension**	**Super-natant**	**Spore suspension**	**Super-natant**	**Spore suspension**
*Alternaria brassicicola*	++	++	++	++	+	++
*Sclerotinia sclerotiorum*	+	+	−	+	−	−
*Verticillium longisporum*	++	++	−	−	−	++

*‘−’– no inhibition; ‘+’ – zones of inhibition are on average between 3 and 5 mm; ‘++’ – zones of inhibition are on average between 6 and 10 mm; experiments were repeated three times.*

UCMB5007 and UCMB5113 showed a wide range of antagonistic activity against most of the bacterial test-cultures. Zones of growth inhibition produced by UCMB5007 were on average twofold larger than those produced by UCMB5113 (Table 2). UCMB5044 demonstrated weak or no antagonistic activity under these experimental conditions.

**FIGURE 2:**
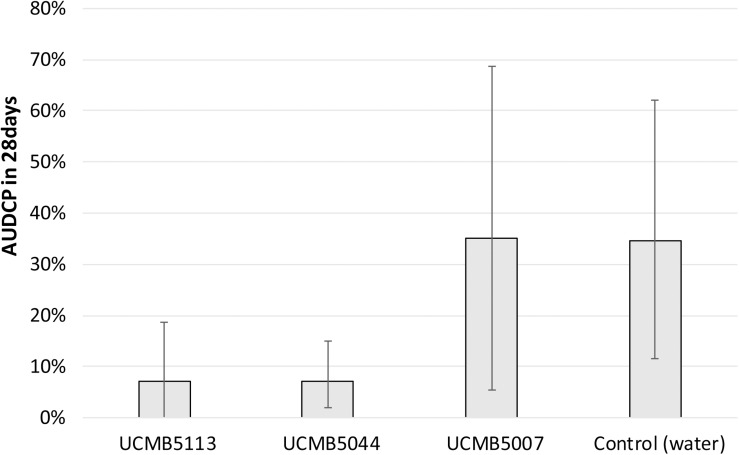
Development of wilting symptoms in infected tomato plants treated with *Bacillus* spores and with water as positive control. AUDCP values were presented as percentage of the maximal AUDCP reported for the positive control plants. Vertical bars depict the standard deviation of AUDCP values estimated in three repeats. Lower AUDCP values correspond to a better protection of plants from the pathogen by the *B. velezensis* strains.

Plant prevention from soil-born fungal pathogens by the treatment of seeds with *Bacillus* spore suspensions followed by inoculation of the bacterial pathogen *R. solanacearum* was performed on tomato seedlings as model plants. Prevention of the tomato wilt disease progression in treated and control plants is illustrated in Figure 2. Areas Under the Disease Progress Curve (AUDPC) were calculated by indices of the disease progression (see Materials and methods). The indices of the disease symptoms were recorded weekly as shown in Supplementary Table 1.

The treatment with UCMB5113 and UCMB5004 largely alleviated the wilt symptoms in the inoculated plants. However, treatment with UCMB5007 had no effect in this assay despite the strong inhibition of the pathogen recorded *in vitro* (Table 2).

### Complete Genome Assembly and Pangenomic Study

The complete genomes of UCMB5007 and UCMB5044 were assembled from genomic DNA reads, thereby resulting in single genome contigs with no gaps or significant ambiguities. In UCMB5007, one problematic area, associated with tandem repeats of the *rrn* operons for ribosomal RNA, was patched by a homologous fragment from UCMB5044. The sequence integrity was then evaluated by a recurring alignment of paired-end Illumina and PacBio reads against the UCMB5007 genome sequence. The At1 genome sequence was assembled into several contigs but with disruptions at the *rrn* operon repeats. The same approach of homologous fragment patching of gaps was followed, but with fragments taken from the UCMB5113 genome, prior to read re-alignment and finalization of the genome sequence. The length of the chromosomes of UCMB5007, UCMB5044 and At1 was 3,983,323, 3,983,303, and 3,888,990 bp, respectively. BUSCO analyses confirmed that the completeness of the final assemblies as satisfactory. The genomes obtained were annotated with the RAST Server and then manually curated to ensure consistent naming of orthologous genes in all genomes. Gene orthology was predicted by OrthoFinder. The annotated genomes were deposited at GenBank NCBI under the accession numbers shown in Table 1. No plasmids were identified in any of these genomes.

**FIGURE 3:**
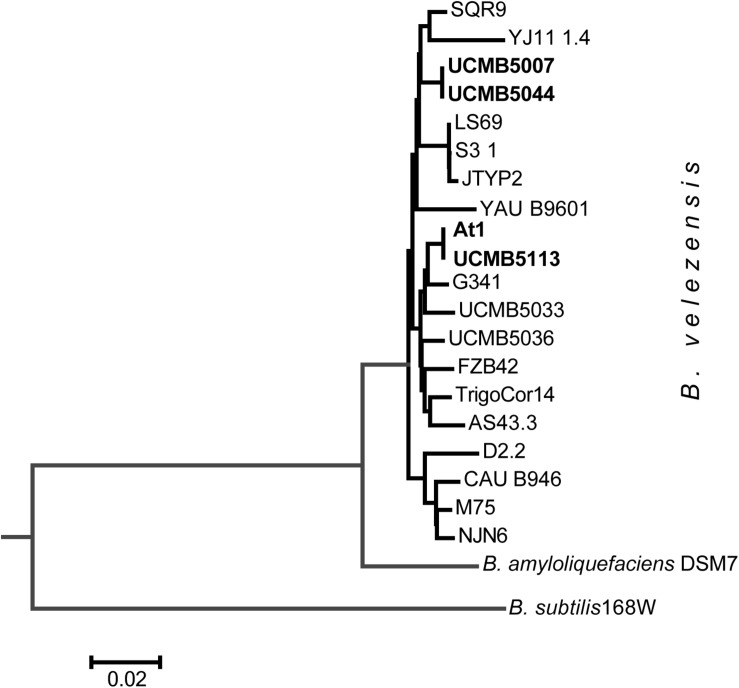
Neighbor-joining phylogenetic tree of newly sequenced *B. velezensis* strains highlighted by bold typeface and the available reference sequences from Genbank NCBI database.

A striking finding was that despite of the obvious differences in their phenotypes, the genomes of UCMB5007 and UCMB5044 were almost identical with only a few polymorphic sites and indels. The number of predicted protein coding genes was the same. In UCMB5007, only 2 genes, adenine deaminase *yerA* and sensor histidine kinase *comP*, were found to have their 5′-ends truncated by frameshift mutations. Despite these frameshift mutations, the transcription levels of these genes were four-fold higher in UCMB5007 compared to UCMB5044. Phylogenetically, strains UCMB5007 and UCMB5044 are quite distant from the central *B. velezensis* cluster comprising of the type strain FZB42, as well as At1 and UCMB5113, amongst others (Figure 3). Complete genome sequencing confirmed the close relatedness between UCMB5113 and At1 as expected from the similarity of their phenotypes. They shared the same protein coding genes, with the exception of a few hypothetical proteins and unexpected stop codons identified in the middle two At1 genes: the fengycin biosynthesis gene *fenD* (i.e., *fenD1* and *fenD2*) and the difficidin biosynthetic gene *dfnG* (i.e., *dfnG1* and *dfnG2*).

The OrthoFinder program revealed 3,453 orthologous protein coding sequences shared by these four strains. Approximately 200 other accessory genes present either in UCMB5113/At1 or in UCMB5007/UCMB5044, were also identified. Many of these genes were predicted as hypotheticals and included fragmented and transcriptionally silent phage proteins. Only a smaller fraction of the accessory genes was functional. Several genes in UCMB5007 and UCMB5044 were functional paralogs present in UCMB5113 and At1 in one copy only. One such duplication in UCMB5007/UCMB5044 involved the *nrdIEF* operon serving in pyrimidine biosynthesis. There were also several paralogous response regulators, ABC transporters and cell-wall binding proteins.

A type I restriction-modification DNA-methyltransferases *hsdMSR* insertion was found in UCMB5113/At1, while an alternative methyltransferase, *Bam*HIM, coupled with the restriction enzyme *Bam*HI, was uniquely identified in UCMB5007/UCMB5044. Another large insertion, a non-ribosomal polypeptide synthetase cluster of 12 genes encoding an unknown polypeptide, was also identified in UCMB5113 and At1. A similar gene cluster, with 99.7% DNA identity, was BLASTN identified in the *B. amyloliquefaciens* type strain DSM7 and in three other *B. amyloliquefaciens* strains. Another operon, BASU_0667-BASU_0674, was uniquely found in UCMB5113/At1 and is putatively involved in fatty acid biosynthesis.

### Synthesis of Polyketides and Lipopeptides

*Bacillus velezensis* strains are equipped with numerous NRPS and polyketide synthases (PKS). Their products are directly involved in bacterial and fungal antagonism (Borriss et al., 2011, 2019; Rabbee et al., 2019). There are eight large gene clusters encoding lipopeptides, siderophores and polyketides: bacillomycin, fengycin, surfactin, bacillibactin, bacillaene, difficidin, macrolactin and bacilysin. UCMB5113 and At1 contain an additional NRPS gene cluster expressed in UCMB5113 as a single operon comprising *nrpsABCD* and *mdtABC* genes. The final product of this biosynthetic gene cluster is yet unknown. A gene cluster similar to *nrpsABCD*/*mdtABC* is present in several *B. amyloliquefaciens* strains (DSM7, SRCM101267, HK1 and GFP2). The NRPS biosynthetic operons encoding the production of surfactin, macrolactin, bacillaene, fengycin and difficidin are strongly expressed in UCMB5113 (Table 4). Interestingly, the mass spectrometric analysis of lipopeptides produced by UCMB5113 confirmed the presence of an unknown linear fengycin species contributing to the antifungal activity of this strain (Asari et al., 2017b). However, it was not clarified by which genes it is encoded.

**TABLE 4:** Average expression of genes of NRPS operons encoding polypeptide antibiotics and regulation of these genes under the effect of maize root exudates.

		**UCMB5113**			**UCMB5007**			**UCMB5044**		
**Locus tag**	**Gene**	**Location**	**Mean^∗^**	**FCh^†^**	**Location**	**Mean**	**FCh**	**Location**	**Mean**	**FCh**
			
**Surfactin**		**340803-366962**			**334732-360891**			**334728-360887**		
BASU_0323	*srfAA*	340803-351558	2351	–3.33	334732-345487	158	1.10	334728-345483	70	–1.58
BASU_0324	*srfAB*	351579-362340	5032	–3.22	345508-356269	333	1.09	345504-356265	110	–2.33
BASU_0325	*srfAC*	362374-366211	2571	–3.14	356303-360140	182	–1.03	356299-360136	79	–1.64
BASU_0326	*srfAD*	366230-366962	438	–2.08	360159-360891	62	–1.14	360155-360887	50	–1.09

**Macrolactin**		**1395814-1449068**			**1380771-1434019**			**1380746-1433994**		

BASU_1395	*mlnA*	1395814-1398121	403	–2.25	1380771-1383078	521	–1.04	1380746-1383053	391	–1.61
BASU_1396	*mlnB*	1398142-1410400	2419	–3.15	1383099-1395360	3446	–1.02	1383074-1395335	1500	–1.61
BASU_1397	*mlnC*	1410399-1415172	1476	–2.33	1395359-1400123	2908	–1.07	1395334-1400098	1504	–1.72
BASU_1398	*mlnD*	1415219-1423928	1836	–3.16	1400170-1408879	3463	1.00	1400145-1408854	1309	–1.44
BASU_1399	*mlnE*	1423921-1430925	3465	–2.85	1408871-1415876	3630	–1.03	1408846-1415851	1695	–1.47
BASU_1400	*mlnF*	1430948-1436660	777	–3.02	1415899-1421611	1387	–1.03	1415865-1421586	370	–1.59
BASU_1401	*mlnG*	1436660-1444042	2522	–2.88	1421607-1428993	2663	1.06	1421582-1428968	805	–1.56
BASU_1402	*mlnH*	1444092-1447944	1057	–2.89	1429043-1432895	968	–1.12	1429018-1432870	425	–1.61
BASU_1403	*mlnI*	1447976-1449068	821	–2.42	1432927-1434019	438	–1.12	1432903-1433994	823	–1.96

**Bacillaen**		**1704004-1776477**			**1686687-1759147**			**1686662-1759122**		

BASU_1650	*baeB*	1704004-1704697	10	2.06	1686687-1687380	66	1.02	1686662-1687355	38	3.31
BASU_1651	*baeC*	1705011-1705881	129	2.33	1687694-1688564	296	1.10	1687669-1688539	664	2.36
BASU_1652	*baeD*	1706017-1706992	89	–1.72	1688700-1689675	399	–1.20	1688675-1689650	222	–1.25
BASU_1653	*baeE*	1706993-1709234	158	–2.26	1689676-1691917	716	–1.22	1689651-1691892	838	–1.31
BASU_1654	*acpK*	1709299-1709548	13	–1.56	1691982-1692231	69	–1.27	1691957-1692206	36	–2.64
BASU_1655	*baeG*	1709599-1710862	61	–3.68	1692282-1693545	269	–1.06	1692257-1693520	115	–1.93
BASU_1656	*baeH*	1710849-1711632	46	–2.89	1693541-1694315	215	–1.07	1693516-1694290	84	–1.54
BASU_1657	*baeI*	1711641-1712391	36	–2.93	1694324-1695074	170	–1.09	1694299-1695049	59	–2.47
BASU_1658	*baeJ*	1712430-1727385	2616	–3.74	1695113-1710062	6909	1.16	1695088-1710037	2589	–1.81
BASU_1659	*baeL*	1727386-1740814	3176	–3.42	1710063-1723482	5919	1.13	1710038-1723457	1992	–1.88
BASU_1660	*baeM*	1740810-1751367	1789	–3.35	1723499-1734035	2739	1.12	1723474-1734010	1343	–1.70
BASU_1661	*baeN*	1751356-1767658	4544	–3.44	1734024-1750329	3338	1.09	1733999-1750304	2247	–1.97
BASU_1662	*baeR*	1767671-1775129	3364	–3.91	1750342-1757800	1244	–1.08		2459	–1.75
BASU_1663	*baeS*	1775265-1776477	50	–2.82	1757935-1759147	122	1.26		199	–1.61

**Bacillomycin**		**1874082-1911365**			**1855158-1892408**			**1855132-1892382**		

BASU_1771	*bmyC*	1874082-1881942	76	–2.84	1855158-1862823	44	–1.02	1855132-1862797	42	–1.26
BASU_1772	*bmyB*	1882025-1898150	143	–3.0	1863101-1879193	49	1.11	1863075-1879167	47	1.13
BASU_1773	*bmyA*	1898194-1910143	154	–2.96	1879237-1891186	27	–1.13	1879211-1891160	36	–1.45
BASU_1774	*bmyD*	1910162-1911365	10	1.61	1891205-1892408	4	2.68	1891179-1892382	14	1.05

**Fengycin**		**1934261-1971931**			**1915318-1952995**			**1915292-1952969**		

BASU_1797	*fenE*	1934261-1938065	485	–3.22	1915318-1919122	72	1.01	1915292-1919096	474	–2.10
BASU_1798	*fenD*	1938083-1948859	922	–3.53	1919141-1929923	173	–1.04	1919115-1929897	1435	–1.78
BASU_1799	*fenC*	1948884-1956534	596	–3.85	1929948-1937598	157	–1.00	1929922-1937572	1312	–2.13
BASU_1800	*fenB*	1956549-1964247	618	–3.52	1937613-1945311	212	1.01	1937587-1945285	1434	–2.10
BASU_1801	*fenA*	1964272-1971931	563	–2.94	1945336-1952995	384	1.26	1945310-1952969	2121	–1.18

**Difficidin**		**2260809-2330328**			**2298518-2368048**			**2298493-2368023**		

BASU_2120	*dfnM*	2260809-2261556	242	–3.38	2298518-2299265	8	–1.14	2298493-2299240	44	–1.48
BASU_2121	*dfnL*	2261605-2262913	245	–3.03	2299324-2300572	10	–1.66	2299299-2300547	69	–1.66
BASU_2122	*dfnK*	2262909-2264064	771	–2.65	2300629-2301784	32	–1.53	2300604-2301759	200	–2.21
BASU_2123	*dfnJ*	2264145-2270361	2408	–3.67	2301865-2308081	153	–1.03	2301840-2308056	480	–1.67
BASU_2124	*dfnI*	2270357-2276510	2194	–3.09	2308077-2314233	133	1.12	2308052-2314208	455	–1.91
BASU_2125	*dfnH*	2276532-2284251	2908	–3.25	2314255-2321974	182	–1.02	2314230-2321949	560	–1.70
BASU_2126	*dfnG*	2284255-2299873	5935	–3.27	2321978-2337593	522	1.15	2321953-2337568	1076	–1.83
BASU_2127	*dfnF*	2299924-2305651	2014	–3.52	2337644-2343371	213	–1.04	2337619-2343346	451	–2.06
BASU_2128	*dfnE*	2305690-2311987	2715	–3.15	2343410-2349707	320	1.04	2343385-2349682	563	–1.89
BASU_2129	*dfnD*	2312005-2324599	5035	–3.46	2349725-2362319	638	1.05	2349700-2362294	1157	–1.89
BASU_2130	*dfnC*	2324638-2325376	170	–2.65	2362358-2363096	44	–1.08	2362333-2363071	69	–2.08
BASU_2131	*dfnB*	2325390-2326755	370	–2.95	2363110-2364475	63	–1.01	2363085-2364450	101	–2.40
BASU_2132	*dfnX*	2326751-2327024	47	–2.01	2364471-2364744	14	–2.43	2364446-2364719	34	–2.20
BASU_2133	*dfnY*	2327048-2328029	126	–3.19	2364768-2365749	34	–1.39	2364743-2365724	97	–2.31
BASU_2134	*dfnA*	2328069-2330328	347	–3.27	2365789-2368048	70	–1.19	2365764-2368023	136	–2.04

***B. amyloliquefaciens* specific polypeptide**		**2508692-2526975**			**Absent**			**Absent**		

	*mdtA*	2508692-2508884	28	–3.02						
	*mdtB*	2508962-2509691	91	–2.23						
BASU_2334	*mdtC*	2509904-2511182	376	–3.51						
BASU_2335	*mdtD*	2511504-2512953	151	–3.47						
mdtE	*nrpsH*	2513139-2513730	115	–3.46						
BASU_2336	*nrpsG*	2513726-2515127	212	–3.33						
BASU_2337	*nrpsF*	2515123-2516446	245	–2.53						
BASU_2338	*nrpsE*	2516442-2521971	1053	–3.21						
	*nrpsD*	2522040-2523231	156	–2.99						
BASU_2340	*nrpsC*	2523505-2524858	188	–2.56						
	*nrpsB*	2524862-2525852	76	–1.48						
	*nrpsA*	2525907-2526975	153	–1.37						

**Bacillibactin**		**3014162-3027137**			**3089364-3102339**			**3089337-3102312**		

BASU_2822	*ybdZ*	3014162-3014378	1	–5.4	3089364-3089580	76	–1.00	3089337-3089553	4	–1.55
BASU_2823	*dhbF*	3014396-3021524	19	–3.51	3089598-3096726	1262	1.03	3089571-3096699	36	–3.10
BASU_2824	*dhbB*	3021538-3022465	3	–1.51	3096740-3097667	222	–1.15	3096713-3097640	7	–2.37
BASU_2825	*dhbE*	3022482-3024108	2	–1.16	3097684-3099310	115	–1.15	3097657-3099283	10	–1.09
BASU_2826	*dhbC*	3024126-3025323	3	3.92	3099328-3100525	30	–1.21	3099301-3100498	4	–2.34
BASU_2827	*dhbA*	3025346-3026132	2	4.29	3100548-3101334	15	1.29	3100521-3101307	3	–4.51
BASU_2828	*besA*	3026267-3027137	2	1.15	3101469-3102339	10	–1.05	3101442-3102312	5	1.52

**Bacillysin**		**3578598-3585293**			**3663282-3669998**			**3663255-3669971**		

BASU_3401	*bacH*	3578598-3579378	37	–2.32	3663282-3664083	146	–1.45	3663255-3664056	29	–2.03
BASU_3402	*bacG*	3579394-3580594	8	–2.56	3664099-3665299	32	–1.12	3664072-3665272	14	–2.10
BASU_3403	*bacE*	3580606-3581788	5	–5.09	3665311-3666493	16	–1.13	3665284-3666466	7	–2.69
BASU_3404	*bacD*	3581784-3583203	14	–1.5	3666489-3667908	43	1.05	3666462-3667881	32	–2.07
BASU_3405	*bacC*	3583220-3583982	3	1.94	3667925-3668687	12	1.47	3667898-3668660	12	–2.35
BASU_3406	*bacB*	3583978-3584692	5	2.18	3668683-3669394	24	1.70	3668656-3669367	22	1.60
BASU_3407	*bacA*	3584678-3585293	5	7.03	3669383-3669998	18	2.56	3669356-3669971	17	4.72
BASU_2844	*ublA*	3040851-3041190	120	–1.06	3116051-3116390	33	–1.08	3116024-3116363	962	–1.47

*^∗^Mean – average values of normalized counts of gene expression on the regular medium and under root exudates exposure experiments repeated 3–5 times; †FCh, fold change of the gene expression under the impact of maize root exudates.*

The level of expression of these genes varied in UCMB5007 and UCMB5044. Expression of the genes involved in the synthesis of macrolactin, bacillaene and difficidin was high in both genomes. Genes of the fengycin encoding operon showed high expression in UCMB5044; and the bacillibactin operon was highly expressed in UCMB5007 (Table 4). The level of expression of surfactin biosynthetic genes was significantly lower in these genomes compared to UCMB5113. In all these genomes, the level of expression of bacillomycin and bacillysin operons was relatively low. There are no gene expression data for At1.

Interestingly, the exposure of all these strains to maize root exudates resulted in no significant effect on the expression of PKS-NRPS genes and in some cases resulted in downregulation. A similar negative effect of root exudates on expression of NRPS genes was reported for *Bacillus atrophaeus* UCMB5137 (Mwita et al., 2016). In contrast to these observations, upregulation of NRPS genes by maize root exudates was reported for FZB42 (Fan et al., 2012; Kierul et al., 2015).

Biosynthesis of lipopeptides, siderophores and polyketides by the selected strains was investigated by MALDI-TOF MS and HPLC-ESI MS. Metabolites identified by MALDI-TOF MS in culture media are shown in Table 5. The strains UCMB5113 and UCMB5007 produced numerous polypeptides and polyketides including bacillibactin, bacillaene, difficidin, fengycin, macrolactin D and surfactin. Bacillomycin D was identified for UCMB5007, but not for the other strains. An unidentified peak was present in the mass spectra of UCMB5113 and At1. No significant lipopeptide or polyketide peaks were identified in the culture medium of UCMB5044. This was not expected as the level of the expression of many NRPS encoding genes was rather high in this strain (see Table 4). Presumably the NRPS synthesis in this strain was blocked post-transcriptionally.

**TABLE 5:** Screening for lipopeptides, siderophores and polyketides by MALDI-TOF-MS.

**Polypeptides**	**UCMB5007**	**UCMB5044**	**UCMB5113**	**At1**
Bacillibactin	+	–	+	+
Bacillaene	+	–	+	+
Bacillomycin D	+	–	–	–
Difficidin	+	–	+	+
Fengycin	+	–	+	+
Macrolactin D	+	–	+	+
Surfactin	+	–	+	+

Mass spectra registered after 24 and 48 h of cultivation of the strains UCMB5007, UCMB5044, UCMB5113, At1 and the reference strain FZB42 are shown in Supplementary Figures 1–5, respectively. They generally corroborated the MALDI-TOF MS results discussed above (Table 6). In this study, the biosynthetic activity of the selected strains was compared to the profile of polypeptides synthesized by the type strain *B. velezensis* FZB42. This strain synthesizes a wide range of secondary metabolites. After 24 h of cultivation, the areas of identified peaks were proportional for all the recorded polypeptides and polyketides synthesized by FZB42, except for bacillaene that declined in the next 24 h of cultivation. In the first day of cultivation, the strains UCMB5113, At1 and UCMB5007 synthesized predominantly macrolactin D and difficidin. The peak areas corresponding to these antibiotics were larger than those for FZB42. UCMB5113 also produced bacillomycin D, while UCMB5007 and At1 produced bacillaene but in lower amounts compared to FZB42. Synthesis of all polypeptides, except for bacillomycin D, was found to be increased after 48 h of cultivation of UCMB5113, At1 and UCMB5007. However, the synthesis of antibiotics in these strains in general remained biased toward macrolactin D and difficidin. It is important to note that the biosynthetic activity of UCMB5044 was comparably weak or absent.

**TABLE 6:** Peak areas retrieved from HPLC chromatograms in culture media after 24 and 48 h cultivation.

**Product^∗^**		**Retention time of peaks (min)**	**Bacterial strains**				
			**UCMB5113**	**At1**	**UCMB5007**	**UCMB5044**	**FZB42**
**24 h cultivation**							
mln		6.29	776	1229.4	413	50	294
		7.55	4049	3874	940	0	462
bmy	D1	6.58	0	0	0	0	324
	D3	6.89	286	0	0	0	467
fen		7.1	97	92.8	0	0	189
dfn		8.76	1210	1247	558	0	471
bae	A	6.52	0	0	131	396	321
		6.59	0	0	130	0	0
		6.75	0	0	278	0	110
		6.84	0	177	0	0	652
	B	7.07	0	0	0	0	280
		7.7	0	0	0	0	0
**48 h cultivation**							
mln		6.29	3349	3658	3064	153	526
		7.55	4864	4417	1273	396	311
bmy	D1	6.58	0	0	0	0	932
bmy	D3	6.89	0	0	32	0	921
fen		7.1	178	185	219	0	270
dfn		8.76	2046	2032	1895	0	1037
bae	A	6.52	178	308	0	0	50
		6.59	178	0	381	0	0
		6.75	144	138	134	0	0
		6.84	285	244	128	0	599
	B	7.07	0	0	98	0	266
		7.7	599	0	198	0	106

*^∗^Product abbreviations: bae, bacillaene; bmy, bacillomycin D; dfn, difficidin; fen, fengycin; mln, macrolactin D.*

In general, the levels of synthesized polypeptide antibiotics were consistent with the patterns of antagonistic activity of these strains (Tables 2, 3). The strain UCMB5044 showed weak or no inhibition of most bacterial and fungal test-cultures. Peak areas of the detected antibiotics were larger in UCMB5113 compared to UCMB5007, but the latter strain produced larger zones of growth inhibition. Cells of UCMB5113 are surrounded by thick and sticky polysaccharide capsules uncommon for other strains of this species (Reva et al., 2004). Thick layers of polysaccharides may reduce the mobility of the synthesized antibiotics and thus decreases the size of the growth inhibition zones.

In the strain At1, MALDI-TOF MS and HPLC showed a pattern of the synthesis of polypeptide antibiotics similar to that of UCMB5113, despite the fact that mutations were detected in the genes *fenD* and *dfnG*, implying that shorter fragments of these genes in the strain At1 remained functional.

### Differential Gene Expression of Orthologous Genes in Different Strains Under the Negative Control Condition

UCMB5007, UCMB5044, and UCMB5113 total RNA samples were obtained from overnight bacterial cultures re-suspended in sterile water as negative controls (2–3 repeats per strain) and in maize root exudates as treated samples (5 repeats per strain).

**FIGURE 4:**
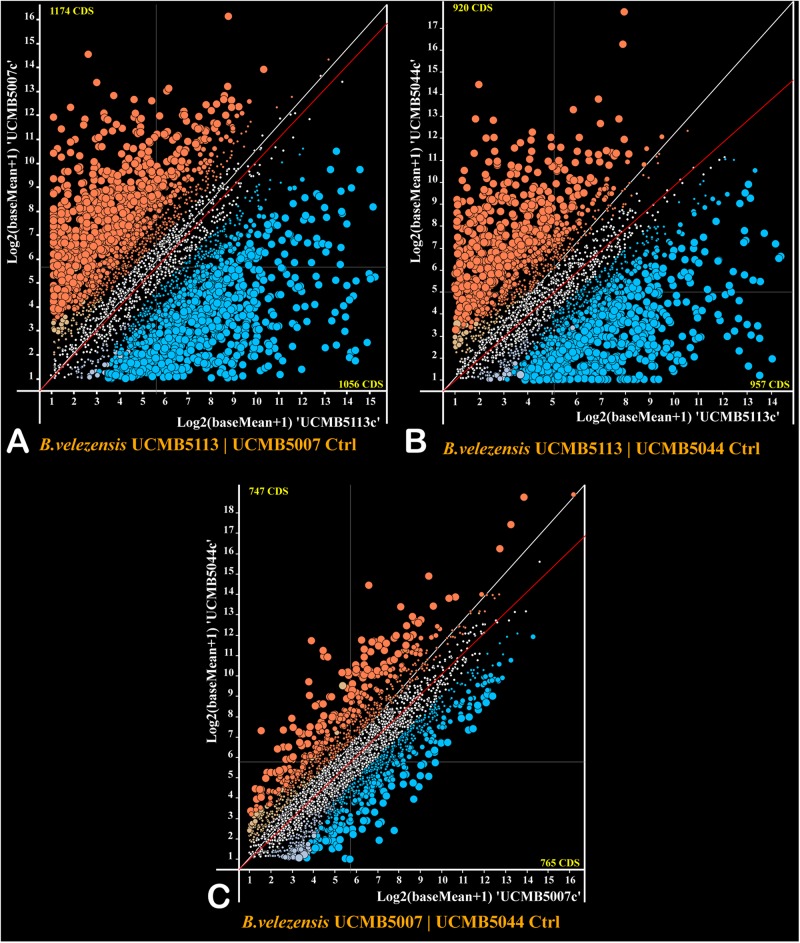
Plots of expression counts calculated for pairs of the strains under negative control conditions with **(A)** UCMB5113 and UCMB5007, **(B)** UCMB5113 and UCMB5044, **(C)** UCMB5007 and UCMB5044 being compared. Every dot on the plots corresponds to a pair of orthologous genes in two genomes. Colored dots depict genes with statistically supported alterations in expression counts, with blue dots applied for genes over-expressed in the *X*-axis genome and red dots showing the over-expression of the genes in the *Y*-axis genome. Transcript abundance differences above 4- and 8-folds change are depicted by increasingly larger dot sizes. Crossed hair-lines indicate the average expression count for both genomes. Deviation of the red trend line from the white diagonal line toward a specific genome axes indicates the organism with the higher overall expression level and likely the higher growth rate.

The program DESeq2 was used to compare normalized gene expression counts between strains estimated for every gene under negative control conditions and represented as scatter plots (Figure 4). The gene expression pattern of UCMB5113 was strikingly different from the gene expression patterns of UCMB5007 and UCMB5044 (Figures 4A,B), with the Pearson coefficients of gene co-regulation 0.182 and 0.167 respectively – which are close to a random distribution. In contrast, the comparison of the gene expression patterns between UCMB5007 and UCMB5044 showed a relatively high level of co-regulation with a Pearson correlation 0.837 (Figure 4C). The most characteristic feature for UCMB5113 was the extremely high level of expression for several genes involved in NRPS synthesis of polypeptides, in particular the genes encoding subunits of surfactin, bacillaene and difficidin synthetase (see also Table 4). Additionally, many genes encoding for fermentation, aerobic respiration and nucleotide degradation enzymes were also upregulated in UCMB5113 when compared to both UCMB5007 and UCMB5044. The latter two strains differed from UCMB5113 in the upregulation of multiple genes involved in biosynthesis of cell wall fatty-acids and lipids.

Two variants of glyceraldehyde-3-phosphate dehydrogenase play a central role in the glycolytic pathway of *Bacillus*, with GapA (*gapA*) acting as a glycolytic enzyme and GapB (*gapB*) catalyzing the reversed gluconeogenesis reaction (Fillinger et al., 2000). The level of *gapA* expression was up to 1,000 fold higher in UCMB5113 than in the other strains, with *gapB* expressed 20–30 fold higher. The *gapA*/*gapB* expression ratio in UCMB5113 is 3.2, with the ratio for UCMB5007 and UCMB5044 around 0.1. This suggests a prevalence of the energy producing glycolytic activity in UCMB5113, while the catabolic gluconeogenesis pathway prevailed in UCMB5007 and UCMB5044 under the control condition.

A peculiarity of the UCMB5113 and At1 phenotypes is the bright orange pigmentation of their colonies. The structure or the biosynthesis of the pigments has not been studied yet. The gene *yisP* encoding phytoene synthase is the central enzyme of the carotenoid biosynthesis in *Bacillus* (López and Kolter, 2010). The level of expression of this gene in UCMB5113 was three-fold lower than in unpigmented UCMB5007 and UCMB5044, which shows the insignificance of this carotenoid biosynthetic pathway in the pigmentation of UCMB5113 colonies. A moderate level of expression of *yisP* in UCMB5113 was determined in other studies that used the RT-PCR approach (M. A. Kharkhota, personal communication).

Reddish pigmentation in bacteria of the *Bacillus subtilis* group may also be associated with the synthesis of pulcherrimin – a red extracellular pigment formed by cyclization of two leucine molecules. Cyclo-dipeptide creates coordinated links with two iron atoms rendering the red color. The pulcherrimin biosynthetic pathway in *B. subtilis* involves activities of a cyclo-L-leucyl-L-leucyl dipeptide cytochrome oxidase CypX and cyclodipeptide synthase PchC (Tang et al., 2006). None of these genes were found in any sequenced *B. velezensis* genome, including UCMB5113 and At1. Moreover, an extraction of pulcherrimin with KOH in methanol showed absence of this pigment in UCMB5113 (Supplementary Figure 6).

In both pigmented strains, UCMB5113 and At1, one uncharacterized NRPS gene cluster was found that might be a candidate for the yet unknown pigment biosynthesis. The gene *nrpsE* of this cluster contains two adenylation domains with unknown substrate specificity implying the final product being built of two amino acid residues. The gene *nrpsE* is highly expressed in UCMB5113 (Table 4). Homologous genes with 69–99% similarity of the encoded protein was identified with BLAST in *B. amyloliquefaciens*, *Halobacillus karajensis*, *Xenorhabdus vietnamensis* and multiple *Pseudomonas chlororaphis*, many of which are also pigmented organisms. However, the role of this NRPS gene cluster has not been studied in any of these bacteria.

In microorganisms of the *B. subtilis* group, synthesis of secondary metabolites is negatively controlled by the AbrB repressor. The expression of *abrB* was 3–7 fold downregulated and the expression of the AbrB repressor, *abbA*, was 50–60 fold upregulated in UCMB5113 compared to UCMB5007 and UCMB5044. This strong suppression of the secondary metabolism repressor may explain the intensive synthesis of multiple secondary metabolites by UCMB5113, particularly the polypeptide antibiotics and polysaccharides. The intensive synthesis of extracellular polysaccharides by this strain may also be associated with the increased expression of the positive regulator SigX, which exceeds the level of the expression in the counterpart genomes by one order of magnitude. Other sigma-factors highly expressed in UCMB5113 are SigH, SigW, SigI and SigL. In contrast, SigF, SigE and SigM were downregulated in this strain compared to UCMB5007/UCMB5044. Upregulated sigma-factors are under negative control of AbrB and under positive control of SigA top-level regulators. The latter one was 16-30 fold upregulated in UCMB5113 as compared to UCMB5007 and UCMB5044.

A high expression level for genes involved in cell growth, cell division and cell wall shaping, i.e., *ftsA*, *ftsE*, *ftsL*, *ftsZ*, *ftsX*, *minC*, *minJ*, *mreB*, and *divIVA*, was also recorded in UCMB5113. Genes for DNA repair enzymes (*recA*, *sodA*, and *radC*) were also activated in UCMB5113, which might be due to a response to the increased growth rate.

Genes with the highest level of expression in UCMB5044, compared to UCMB5007 and UCMB5113, were those involved in amino acid utilization (*rocG*, *artP*, *appC*, *dppC*), utilization of acetoin produced from xylose and other plant associated sugars and polymeric carbohydrates (*acoA*, *bglS*), and in gluconeogenesis (*pckA*).

UCMB5007 expressed several metabolic enzymes involved in pyrimidine biosynthesis at high rates, including an orotate phosphoribosyltransferase (*pyrE*), a dihydroorotate dehydrogenase electron transfer subunit (*pyrK*) and a dihydroorotate dehydrogenase (*pyrD*). Other highly expressed genes were cystathionine gamma-lyase (*mccB*) and L-cystine uptake protein (TcyP) of the cysteine biosynthesis pathway, ribosomal RNA small subunit methyltransferase C involved in ribosomal maturation, an acetolactate synthase (*ilvH*), and a NAD-dependent malic enzyme (*maeA*). It may be concluded that the pyrimidine biosynthesis, cysteine uptake and organic acid utilization were specifically activated in UCMB5007.

### Gene Regulation in Response to Root Exudates

**FIGURE 5:**
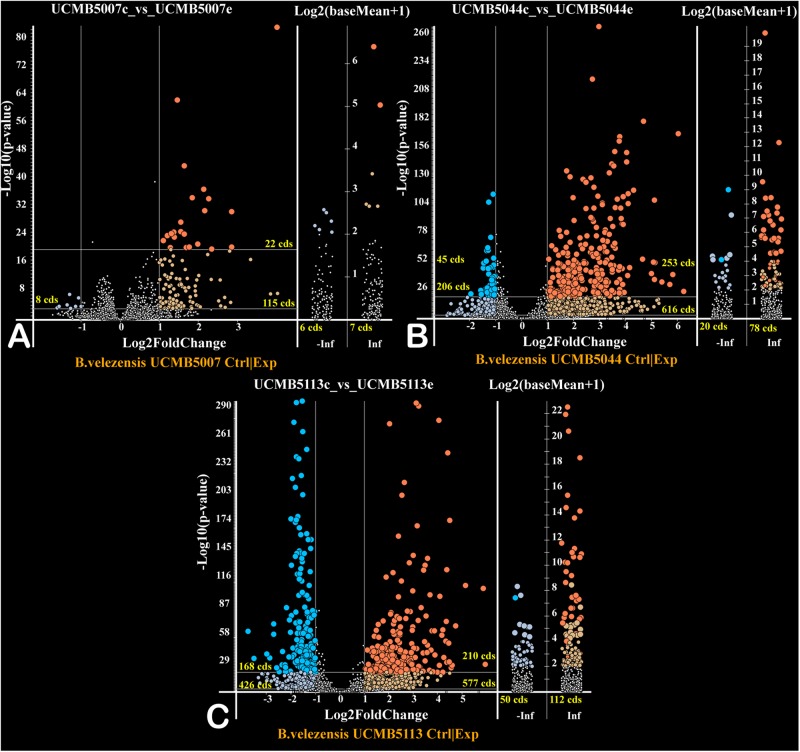
Volcano plots of gene regulation in each strain (**A** – UCMB5007; **B** – UCMB5044; **C** – UCMB5113) after the maize root exudate treatments in comparison to the negative control re-suspension of bacterial cells in water. Dots on the plots depict the negative (blue) and positive (red) gene regulation. Log_2_ fold gene expression changes are on the *X* axes, with the common *p*-values logarithms of gene expression estimations on the *Y* axes. *P*-values were calculated by DESeq2 algorithm on five repeats of gene expression counts under root exudate exposure, and 3 repeats of gene expression counts under the negative control condition. Genes expressed only on root exudates are plotted along the axis of expression counts in the positive infinity columns (Inf), and the genes expressed only in the negative controls in the negative infinity columns (–Inf). Color intensity of dots depicts estimated *p*-values. Numbers of regulated genes of different categories are indicated.

The effect of a 20 min root exudate exposure on gene expression patterns in the bacterial cell suspension of the selected strains was investigated. Volcano plots (Figure 5) show the estimated *p*-values of the differential expressed genes under experimental (with root exudates) and negative control conditions. Genes only expressed at the control condition (−Inf) or experimental condition (Inf) are depicted in the corresponding columns (Figure 5).

**FIGURE 6:**
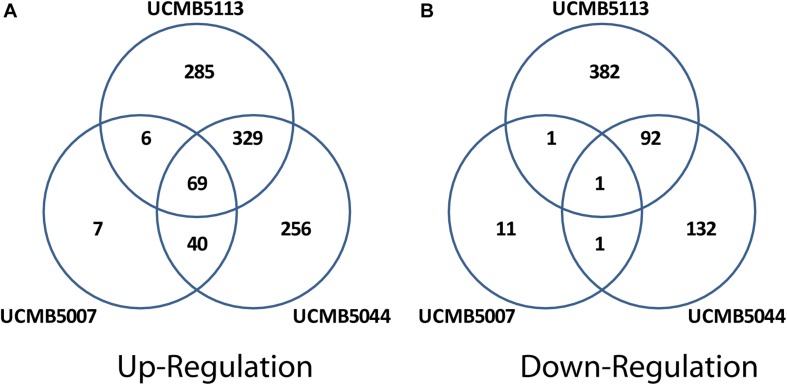
Venn diagrams of positive **(A)** and negative **(B)** regulation of orthologous genes in three *B. velezensis* strains.

Exposure of cells to root exudates affected the expression of many genes in the plant-associated strains, UCMB5113 and UCMB5044, but had only a moderate effect on the transcription of genes in UCMB5007. The latter strain was isolated from calf intestinal micro-flora and is unlikely associated with plants. Venn diagrams (Figure 6) show co-regulation of orthologous genes in different strains under the influence of root exudates. Many orthologs upregulated by root exudates in UCMB5007 were also upregulated in the other two bacteria. These genes most likely represent a pool of a general response of *B. velezensis* to root exudate stimuli. The most activated in all three genomes was the arginine biosynthesis operon, which includes the genes *argB*, *argC*, *argD*, *argG*, *argJ*, and *argH*. This operon showed little or no expression at the negative control condition in all tested bacteria. Transcription of the *yclJK* operon, which encodes an oxygen limitation stress-response two-component regulatory system, was also activated in all the strains. Phage related proteins and transposons were another common group of the activated genes. The increased transcription levels of these phage related genes most likely resulted in a subsequent activatation of protective restriction enzymes, such as *Bam*HI. The latter one was upregulated threefold in UCMB5007 and ninefold in UCMB5044 compared to their negative controls.

Repression of genes by root exudates was strain-specific. The only gene that was strongly downregulated in all three bacteria was ATP:Cob(I)alamin adenosyltransferase encoded by *pduO* (synonym *yvqK*). This enzyme catalyzes the final step of the synthesis of vitamin B12, with its repression most likely caused by providing bacteria with a sufficient amount of the vitamin with root exudates (Vranova et al., 2013).

The root exudates caused a significant re-programing of the global gene regulation in UCMB5044 and UCMB5113, but to much smaller extend in UCMB5007. UCMB5044 and UCMB5113 shared 329 co-activated genes and 92 downregulated genes affected by the root exudate stimuli. These genes include various regulatory proteins and metabolic enzymes showing that the bacterial response to the root exudate stimuli was rather complex. Three-fold upregulation of the swarming motility gene (*swrAA*) observed in both organisms may indicate a preparation for biofilm formation. However, the central chemotaxis genes (*cheA*, *cheB*, and *cheC*) were downregulated in both genomes.

### Patterns of Epigenetic Modifications of Chromosomal DNA

Profiling of epigenetic modifications in sequenced genomes was performed using the SMRT kinetic analysis of IPD ratios assigned to each nucleotide. The program identifies locations of nucleotides causing a delay of base calling during the sequencing and analyses the context information to predict associated sequence motifs and types of modifications. The most common type of epigenetic modifications is methylation of adenosine at 6th nitrogen atom (m6A) and cytosine at 4th nitrogen atom (m4C) (Casadesús and Low, 2006). However, it should be noted that the program SMRT Link does not differentiate between m4C and m5C methylation. The latter can thus not be ruled out. Moreover, SMRT kinetic analysis identifies many other nucleotides which delay the base calling with a statistical reliability owing to modifications of unknown nature. Hereafter, in the text, these unknown modifications to adenosine, guanine, cytosine and thymine are denoted as modA, modG, modC and modT, respectively. The statistical reliability of predicted modified nucleotides is represented by quality value (QV) modification scores. QV scores 14 and 21 correspond to *p*-values 0.05 and 0.01, respectively.

**FIGURE 7:**
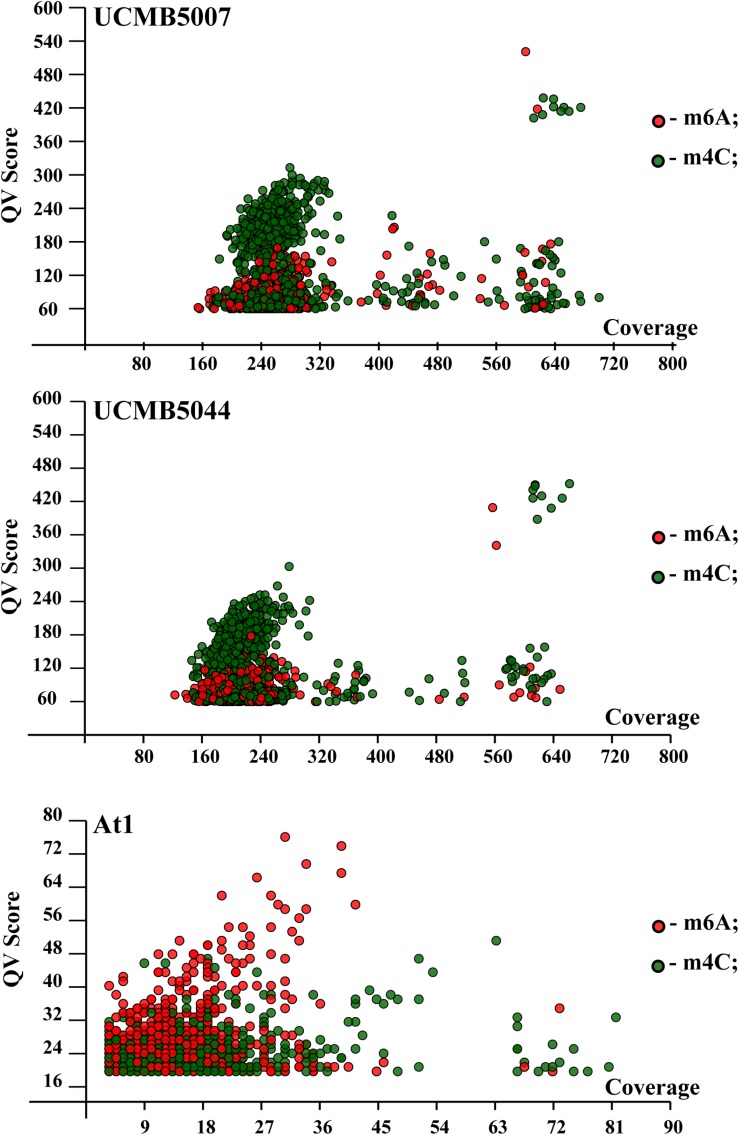
QV modification score *vs*. coverage scatterplots calculated for UCMB5007, UCMB5044 and At1 genomes. Base modification dots are plotted by the corresponding QV scores and coverage values estimated by SMRT Link *ipdSummary* tool based on the alignments of PacBio reads against the reference sequences. Methylation types are denoted by dots of different styles, as shown in the legend. The m4C sites may correspond to both types of cytosine methylation at 4th and 5th carbon atoms.

QV modification scores *vs.* coverage scatterplots of m6A and m4C sites identified in the sequenced genomes are shown in Figure 7. Every dot on this plot corresponds to a methylated adenine (in red) or cytosine (in green) characterized with a QV modification score above or equal to 60. In UCMB5007 and UCMB5044, the dominant type of methylation is m4C. The methylation pattern in At1 was obtained based on a lower coverage of PacBio reads that reduced the number of statistically reliable predictions of modified nucleotides. Nevertheless, it is obvious that in this genome the dominant type of methylation is m6A that is characteristic for the majority of prokaryotes (Casadesús and Low, 2006).

Analysis of DNA sequences flanking modified nucleotides demonstrated a strain-specific distribution of repeated contextual motifs identified by the *motifMaker* algorithm implemented in the SMRT Link package (Table 7).

**TABLE 7:** Motifs and frequencies of nucleotide modifications in three sequenced genomes as predicted by SMRT Link *motifMaker* tool.

**Motif^∗^**	**Type**	**UCMB5007**		**UCMB5044**		**At1**	
		**Modified sites**	**% of found motifs**	**Modified sites**	**% of found motifs**	**Modified sites**	**% of found motifs**
G*G*AT**C**C	m4C^†^	465	99.4%	460	98.3%	No	
VV**A**TGVNYR	m6A	272	1.8%	295	2.0%	23	0.1%
CK**T**ATASYD	modT	48	14.6%	29	8.8%	No	
BNNNNN**T**TTATACY	modT	14	6.7%	13	6.7%	No	
SGGN**T**GAACD	modT	12	4.0%	13	4.4%	1	0.1%
**G**GB	modG	1348	0.5%	2038	1.0%	475	0.2%
GYT**A**NDNNVNN*T*GC	m6A	No		No		66	26.9%
GC**A**DNNNNNN*T*ARC	m6A	No		No		98	27.6%
TNGNNNTGNGTAGNNNN**C**	m4C	No		No		9	90.0%

*^∗^Modified nucleotides are shown in bold and underscored. In the case of bipolar methylation on both strand of DNA, complement nucleotides lying opposite to the modified ones are shown in italic typeface. It should be noted also that the level of methylation of the chromosomal DNA in At1 was underestimated due to significantly lower coverage of PacBio reads compared to two other genomes. ^†^The SMRT Link *motifMaker* tool always indicates methylated cytosine residues as m4C irrespectively of whether methylation occurred at the 4th and 5th carbon atom. In GGATCC sites, cytosines are methylated by the cognate *Bam*HIM methylase at 5th carbon atom (McClelland and Nelson, 1988) and should strictly be denoted as m5C. However, in this paper the m4C notation was used throught the text for consistency with by *motifMaker* predictions.*

Bipolar m4C methylation of palindromic sequences G*G*AT**C**C in UCMB5007 and UCMB5044 is caused by the methyltransferase BamHIM. This methylation prevents DNA cleavage by the type-2 restriction enzyme *Bam*HI. Restriction-modification systems are frequent in bacterial genomes. They provide bacteria with a defense against foreign DNA. The native DNA is methylated and protected in this way against cleavage by the restriction endonucleases, while the DNA of phage and plasmid intruders is destroyed by these enzymes. The genes *bamHI* and *bamHIM* are located on the chromosomes UCMB5007 and UCMB5044 next to each other, but transcribed in opposite directions. Root exudates upregulated *bamHI* expression 4–8 fold, and *bamHIM* 2–4 fold. Next to the restriction-modification genes, there is a fragment of a transposase *tnpC* implying the acquisition of these genes by horizontal gene transfer. Despite some level of fragmentation, *tnpC* is still transcribed in UCMB5007, but is transcriptionally silent in UCMB5044.

In At1, palindromic sequences G*G*AT**C**C are not methylated as this genome does not contain *bamHIM* and *bamHI* genes. Instead, there is an *hsdMSR* operon encoding three sub-units of a methyltransferase. Upstream of this gene cluster, a prophage derived gene *yqcG* is located that may indicate previous horizontal acquisition of this region. These genes are absent in UCMB5007 and UCMB5004. Because of this, it may be assumed that the bipolar m6A methylation of GYT**A**NDNNVNN*T*GC and GC**A**DNNNNNN*T*ARC sequences, identified in At1, occured due to activity of this methyltransferase complex.

The methylation m4C in At1 was generally sporadic except for 9 sites associated with the motif TNGNNNTGNGTAGNNNN**C**. The same motifs, all in non-coding sequences, were found in UCMB5007 and UCMB5044 but without any sign of methylation.

All three genomes displayed another m6A methylation pattern associated with the VV**A**TGVNYR motif, which may be due to an activity of an unidentified methyltransferase shared by these bacteria. The efficacy of the DNA methylation by this methyltransferase was much lower than by the two former enzymatic complexes considered above. Only a small fraction below 2% of available motifs was methylated (Table 7).

**FIGURE 8:**
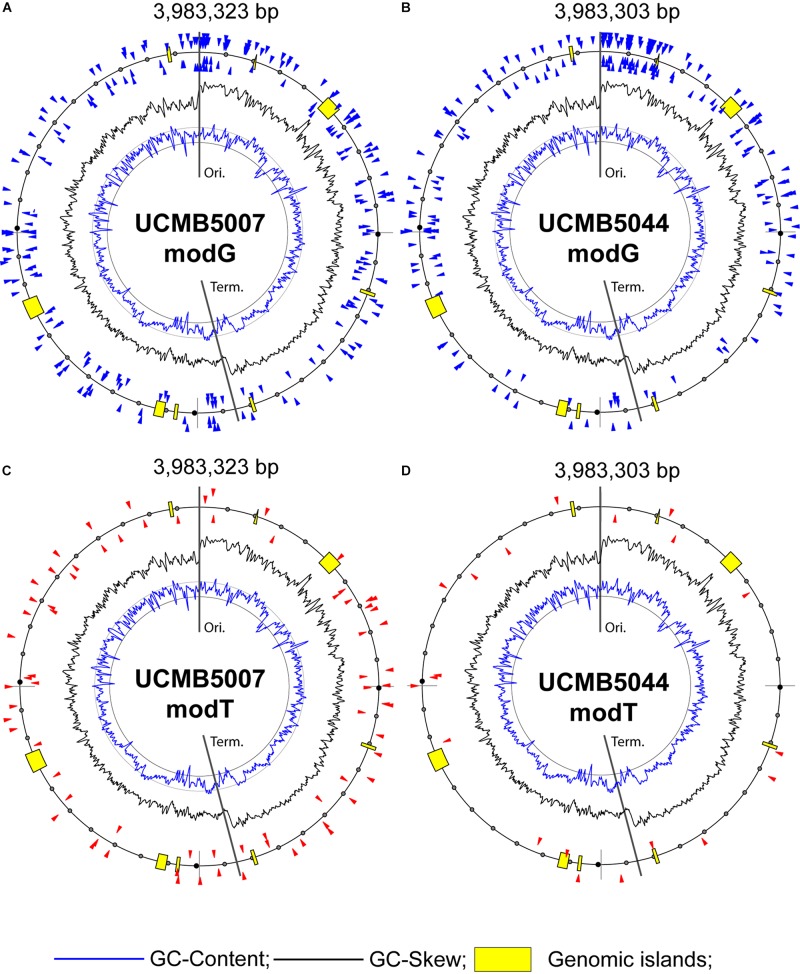
Distribution of modified bases with QV scores ≥ 100: **(A)** modG in UCMB5007, **(B)** modG in UCMB5044, **(C)** modT in UCMB5007 and **(D)** modT in UCMB5044. Blue and black histogram lines show local deviations of GC-Content and GC-Skew in 8 kbp sliding windows with 2 kbp step, respectively. Insertions of horizontally transferred genomic islands (prophages) are depicted by yellow boxes. Chromosomal replication origin (Ori.) and terminus (Term.) are indicated.

The nature of other types of nucleotide modifications denoted as modG and modT is unclear. There were also some modA and modC modifications, but those were not frequent and were sporadically distributed without associations with any sequence motifs. Guanosine and adenosine residues may be oxidized to 8-oxoguanosine and 8-oxoadenosine. Another possible modification, *O*-6-guanine methylation, may be a result of an abnormal activity of DNA methylases. However, the later modification is highly mutagenic and is removed by the 6-*O*-methylguanine-DNA methyltransferase *radA*, which is actively transcribed in all these three genomes. Nothing is known about the possible chemical nature of thymidine modification, although the residues delaying the base calling were abundant in UCMB5007 and UCMB5044. Association of modT and modG sites with rather complex contextual sequences (Table 7) suggests a possible involvement in this process several DNA binding proteins or non-coding regulatory RNA molecules recognizing specific motifs. Only a small fraction of available motifs was affected by these epigenetic modifications making the distribution of modG and modT sites strain-specific. In Figure 8, modified nucleotides on the direct (clockwise) or reverse-complement (counter-clockwise) strands of bacterial chromosomes are depicted by triangle markers located respectively outside or inside of the circular line on the plots.

Epigenetic modifications are known to be involved in gene regulation. This phenomenon has been studied in detail in *Escherichia coli*. One classical example of this type of regulation is the pyelonephritis-associated pili (*pap*) operon in uropathogenic *E. coli* controlled by DNA adenine methylase (Dam). Switching between the ON/OFF states of the *papBA* genes determines the binding of two proteins at two GATC sites, before and after the promoter. The operon is turned to the ON state when methylation occurs proximal to the promoter, and *vice versa* (Hernday et al., 2002). Another example includes the gene *flu*, encoding for the outer membrane protein Ag43 in *E. coli*. This gene is regulated at three GATC sites and its expression is repressed by the oxidative stress response protein, OxyR. Binding of OxyR to a GATC site masks this site, therefore blocking methylation by Dam, and turning expression to the OFF state (Haagmans and van der Woude, 2000).

Acquisition of the *Bam*HI/*Bam*HIM restriction-modification gene cluster by the strains UCMB5007 and UCMB5044 and the absence of the alternative methyltransferase complex *hsdMSR* present in At1 and UCMB5113 have changed the global distribution of methylated nucleotides in these genomes. In contrast to cytosine methylation in eukaryotes, adenine methylation is more frequent in the bacterial world. However, this was not observed in UCMB5007 and UCMB5044. Methylation of nucleotides modulates activities of regulatory DNA-binding proteins (Sánchez-Romero et al., 2015) and frequently causes transcriptional repression rather than activation (Casadesús and Low, 2006). In the latter two genomes, there are 11 GGATCC bipolar methylated sites up-front of transcription start codons of 12 protein coding genes. These modifications can potentially interfere with promoter sequences of these genes and affect their transcription. There are no such methylation sites in At1 and probably not in UCMB5113. Out of these 12 genes, 8 genes were significantly downregulated in UCMB5007 and UCMB5044 compared to UCMB5113 at the negative control condition. These included the sigma-factor *sigA*; oligopeptide transport system permeases *oppB* and *oppC*; bacillaen biosynthetic gene *baeG*; dTDP-4-dehydrorhamnose reductase *spsK*; 5-keto-2-deoxygluconokinase *iolC*; and two hypothetical proteins *ykkB* and *yuxK*. One gene was upregulated: hypothetical protein *ylaH*; and for several genes the expression alteration was statistically insignificant: transcription antitermination protein *nusG*; ribose ABC transport protein *rbsA*; and cytochrome *d* ubiquinol oxidase subunit *cydB*. The most profound effect on the global expression pattern may have been the downregulation of the top-level transcriptional regulator SigA, which regulates activities of many other sigma-factors. A double methylated site **C**TA*G* overlaps -5.-8 nucleotides of the SigA promoter region in UCMB5007 and UCMB5044.

Methylation of restriction sites may be blocked by cognate DNA binding proteins, which prevents both the methylation and the cleavage of DNA by the restriction enzymes (Casadesús and Low, 2006). This may explain the fact that not all GGATCC motifs are methylated in the sequenced genomes (Table 7). In several cases GGATCC motifs were methylated only in one strand. Six GGATCC sites in UCMB5007 and one site in UCMB5044 were methylated only in one strain. Six of these sites were within transcribed sequences and one was located in a non-coding sequence. The effect of these specific methylations on gene transcription regulation remains unclear.

More than 40% of motifs VV**A**TGVNYR were methylated uniquely only in UCMB5007 or in UCMB5044. Eight of these non-homologous sites were in the 5′-end untranslated flanking regions near the start codons of protein coding genes. Compared to UCMB5113 under negative control conditions, the affected genes in these strains were downregulated in five cases while two cases indicated upregulation. Only one case showed insignificant regulation patterns.

Little or nothing is known about modG and modT modifications. The number of modG modifications was similar in UCMB5007 and UCMB5044, but in the latter strain the density of modified sites was higher in the region downstream of the replication origin (Figure 8). The number of high scored modT sites was larger in UCMB5007 than in UCMB5044. These modifications may play a role in silencing of genes. For example, the transposase *tnpC*, next to the *bamHI*/*bamHIM* restriction-modification complex, was transcriptionally silent in UCMB5044 in contrast to the homologous gene in UCMB5007. The only difference between them is the presence of a modT site within the gene body and two sites, modT and modG, on the opposite strand in UCMB5044. However, it may be a coincidence as the statistical analysis of associations of modG and modT sites with alternative gene regulation in these genomes did not reveal any statistically reliable trend. If these modifications are caused by binding cognate regulatory proteins or non-coding RNA, the effect of the binding may be ambivalent depending on properties of the regulatory elements and the precise location of modified sites within recognized sequences. It can be seen in Figure 8 that high scored modG and modT sites avoid horizontally acquired prophages comprising remnants of transcriptionally silent phage related genes. These modifications were more common in coding sequences that implies their involvement in gene regulation.

## Discussion

*Bacillus velezensis* strains are used in agriculture for plant protection and growth promotion as a ‘green’ alternative to chemical biopesticides and fertilizers (Thakore, 2006; Alina et al., 2015). Application of bacteria of the *B. subtilis* and/or *B. velezensis* group in veterinary and medical probiotics is also promising (Sorokulova et al., 2008; Wang et al., 2009; Cutting, 2011; Horosheva et al., 2014; Elshaghabee et al., 2017; Ye et al., 2018). In the current work, four *B. velezensis* strains were evaluated: UCMB5113, isolated from soil but with an outstanding experimentally proven ability to colonize and protect plants; At1, with similar phenotype isolated from seedlings of *A. thaliana*; UCMB5044, isolated from inner tissues of cotton plant stem; and UCMB5007, isolated from calf intestine and previously used as a probiotic agent. The research question was to investigate what the difference between these strains could be, considering their association with different habitats and producing distinct biological traits in terms of their preferential growth conditions, biosynthetic and antimicrobial activities. Affiliation of *Bacillus* isolates with specific habitats is a disputable question because of the extreme resistance of their spores to harsh conditions, although the metabolically active cells may have certain preferences. Isolation of a *Bacillus* strain may indicate good survival capacity of the spores rather than its habitat specificity. For example, the strain UCMB5007 could be a plant colonizer introduced to calf intestines through the ingestion of grass. The pattern of gene regulation in this strain under the root exudate exposure disproved this possibility by showing no inclination of this strain to colonize plants. Most likely the survival strategy of this strain differs significantly from that of UCMB5044 and UCMB5113, which showed a strong positive response to root exudate stimuli. Out of 614 genes in UCMB5113 and 585 genes in UCMB5044 upregulated during 20 min cultivation with maize root exudates, 329 genes were the same orthologs in both genomes (Figure 6A). This result could not be expected considering the identity of the chromosomal sequences of UCMB5007 and UCMB5044, which obviously were distant from UCMB5113, which also showed a distinct gene expression profile when compared at the negative control condition (Figures 4A,B). An intuitive expectation was that two strains of the same species inhabiting the same habitat should be phylogenetic relatives and display similar gene regulation profiles. However, this was not the case with the two plant associated strains, UCMB5113 and UCMB5044, which obviously were distant to each other but responded similarly to the root exudate stimuli in contrast to UCMB5007 (Figure 6). It can be assumed that the ability to recognize environmental signals associated with the plant rhizosphere conferred the strains UCMB5044 and UCMB5113 with the ability to colonize and protect plants (Figure 2). In many other aspects including the phenotype and biosynthetic capacities, the strains UCMB5044 and UCMB5113 were as much different as it could be assumed from the dissimilarity of their gene expression patterns.

This study showed the strains UCMB5113 and At1 to be outstanding producers of polypeptide antibiotics. This biosynthetic activity may result from a significant downregulation of the AbrB repressor. Such an energy wasteful lifestyle makes these strains sensitive to the availability of nutrients in the medium. In contrast to UCMB5007 and UCMB5044, these bacteria grow poorly on the M9 minimal medium – even when the medium was supplemented with root exudates (Figure 1C). This suggests a possible adaptation of these strains to the endophytic or epiphitc environment rich in carbohydrates and vitamins produced by plants.

*Bacillus velezensis* strains forming orange colonies, similar to those of UCMB5113 and At1, were frequently isolated from oilseed rape (*Brassica napus*) cultivated in Sweden, but they were not observed among isolates from cotton plants (*Gossypium* sp.) cultivated in semi-desert areas of Tadzhikistan (unpublished communication by O. N. Reva). Contrary, the later habitat was abundant with *B. velezensis* strains of several morphological phenotypes represented by UCMB5044 and several other distinct morphotypes such as UCMB5033 and UCMB5036 sequenced earlier (Johansson et al., 2014). This observation suggests a geographic separation between plant-associated *B. velezensis* lineages possibly adapted to different climatic zone and/or different plant species.

UCMB5044 is opposite to UCMB5113 and At1 in many aspects. UCMB5044 is an oligotroph, which grows preferentially on nutrient poor media. On LB medium, the growth curve starts to decline early probably due to acidulation of the environment. The synthesis of all secondary metabolites is strictly reduced in UCMB5004, either on the transcriptional, post-transcriptional or translational levels. The strain shows no or little inhibition of test-cultures *in vitro* (Table 1) but effectively controls phytopathogens *in vivo* (Figure 2). This implies that this organism can synthesize the antibiotics and elicitors of plant systemic resistance only in response to specific signals from plants and/or phytopathogens. These considerations also led us to a conclusion that *Bacillus* strains selected for plant protection in Europe may not be active in arid or tropical areas. Local isolates may show better PGPR activities even if their antagonistic activity against phytopathogens *in vitro* is as weak as in the case with UCMB5044. Also, this study showed the inappropriateness of the selection of PGPR strains solely based on their ability to inhibit growth of pathogens *in vitro*. Following this strategy, the strain UCMB5044, with a strong capacity to protect plants against fungal phytopathogens, would be rejected, while the strain UCMB5007 showing no such capacities, would be selected as the most promising one (see Figure 2).

The strain UCMB5007 represents the copiotrophic lifestyle. However, it is not so much dependent on the nutrient rich environment as UCMB5113 and At1. UCMB5007 produces a large variety of secondary metabolites and showed the best inhibition of various pathogens *in vitro*, including phytopathogens, but it cannot protect plants *in vivo* due to its inability to colonize the rhizosphere and plant tissues. The strain was isolated from cow intestinal microflora and was used successfully as a probiotic to control diarrhea in calves. However, currently there are no data to claim that this strain is a representative of the normal gut microflora of ruminants. Despite of the distinction in their phenotypes and lifestyle, the genome sequences of UCMB5007 and UCMB5044 were almost identical implying a crucial role of epigenetic modifications in the evolutionary adaptation of these strains.

For a long time, bacterial evolution was considered as being driven exclusively by sequence alterations caused by local mutations, deletions and insertions of horizontally transferred elements creating genetic variations (Hershberg, 2015). Today, the advance in sequencing technologies allows sequencing and comparison of genomes of multiple bacterial strains isolated from a wide variety of habitats and exploiting different survival strategies. Recent studies provided a lot of evidences to consider the micro-evolution of bacteria as stable in time but with potential to undergo reversible phase variations rather than being limited by a hard engraving into genome sequences.

Bacteria are constantly faced with the challenge to maintain their fitness within changing environments under various biotic and abiotic stresses. In response to these stresses, bacteria may alter their phenotypes rapidly by modulating gene expression patterns through phase variations. Phase variation is a heritable but potentially reversible process that can help bacteria in rapid adaptation to changing environments. It is a form of gene regulation that involves altering between low and high levels of gene expression leading to diversification of bacterial populations in a relatively short period of time. Various molecular mechanisms influence the phase variations, either genetically, involving changes in the DNA sequence, or epigenetically, involving the methylation of DNA at specific loci (van der Woude and Bäumler, 2004). DNA methylation enables bacteria to control epigenetically the reversible ON/OFF switching of important genes (Henderson et al., 1999).

*Bacillus velezensis* strains selected for this study are optimal models to study adaptive phase variations and to translate this knowledge into practical application. These bacteria are generally resistant to significant genomic rearrangements and horizontal genes exchange. The core genome constitutes a large part of the coding sequences (Figure 6), leaving little room for accessory elements represented mostly by transcriptionally silent debris of prophages. Despite sharing similar genes, gene expression profiles were found strikingly different in these bacteria (Figure 4). The differential gene regulation may be caused by alterations in global methylation patterns due to acquisition of alternative restriction-modification genes such as *Bam*HI/BamHIM complex in UCMB5007/UCMB5044 and the DNA-methyltransferase operon *hsdMSR* in At1/UCMB5113. A gene BASU_0597 in UCMB5113 located upstream of the *hsdMSR* operon was predicted as a truncated restriction enzyme. No transcription from this gene was observed. It may be concluded that this horizontally acquired restriction-modification complex retains only the DNA methylation activity, which may be of importance for gene regulation in UCMB5113.

Further adaptation of UCMB5007 and UCMB5044 to different habitats was also most likely facilitated through epigenetic variations driven by yet unclear mechanisms balancing regulatory elements’ activities, as reflected in the genomes in the form of specific modG and modT epigenetic modifications.

In terms of the effective application of bacterial strains for biocontrol of plant diseases and in probiotics, it can be concluded that the adaptive gene regulation in response to environmental signals, controlled by phase variations, should play larger roles during the selection of appropriate bioactive strains rather than the phylogenetic relatedness, gene repertoire, ability to grow up on root exudates as a sole source of nutrients or a strong antagonistic activities observed *in vitro*. The aim of this research was to introduce new model strains that comprehensively characterized by bioactivity spectra and genetic, transcriptional and epigenetic profiles to act as benchmarks for further studies. These strains were not yet intensively tested under field conditions and cannot be recommended at the current stage for any plant protection or biocontrol application. Field trials will be a subsequent step to further prioritize the selected strains and optimize efficacy for different crops and conditions. However, it seems clear that beneficial microorganisms will play important roles in future agriculture for crop production, integrated pest management and animal health.

## Data Availability Statement

The datasets generated for this study can be found in the NCBI: PRJNA176687, CP041143.1, PRJNA548267, CP041144.1, PRJEB1418, HG328254.1, PRJNA176703, CP041145.1.

## Author Contributions

OR was involved in funding acquisition and conducted DNA and RNA sequencing, genome assembly, annotation, epigenetic profiling, and manuscript writing and editing. DS conducted root exudate assays, RNA sequencing, and manuscript writing and editing. LM performed PGPR assays and growth curves calculation. AD was involved in running PGPR assays. DM conducted genome annotation and manuscript editing. MJ performed gene expression profiling and manuscript editing. WC conducted mass spectrometry studies and manuscript editing. StL conducted PacBio and Illumina MiSeq sequencing, genome assembly, and manuscript editing. CA was involved in funding acquisition, PacBio and Illumina MiSeq sequencing, genome assembly, and manuscript editing. LA was responsible for providing bacterial strains from the UCMB culture collection and for manuscript editing. MK conducted bacterial pigment extraction and analysis, and manuscript editing. DT supervised the conducting of PGPR activity assays and participated in manuscript editing and raising of funds. SyL supervised the conducting of PGPR activity assays and participated in manuscript editing and raising of funds. JV conducted mass spectroscopic measurements and manuscript editing. RB was involved in mass spectrometry study supervision and manuscript editing. JM supervised the conducting of PGPR activity assays and participated in manuscript editing and raising of funds.

## Conflict of Interest

The authors declare that the research was conducted in the absence of any commercial or financial relationships that could be construed as a potential conflict of interest.

## Footnotes

1 http://rast.nmpdr.org

2 https://www.sanger.ac.uk/science/tools/artemis

3 http://nrps.igs.umaryland.edu/

4 https://www.pacb.com

## Abbreviations

AUDPC: Areas Under Disease Progression Curve
bae: bacillaene
bmy: bacillomycin
dif: difficidin
fen: fengycin
HPLC: High-Performance Liquid Chromatography
LB: Luria broth nutrient medium
m4C: *N*-methylcytosine
m5C: 5-methylcytosine
m6A: N^6^-methyladenosine
MALDI-TOF MS: Matrix-Assisted Laser Desorption/Ionization (MALDI) Time-Of-Flight (TOF) Mass Spectrometry (MS)
mln: macrolactin
modA: epigenetically modified adenosine
modC: epigenetically modified cytosine
modG: epigenetically modified guanosine
modT: epigenetically modified thymidine
NCBI: National Center for Biotechnology Information
OD: optical density
PacBio: Pacific Bioscience technology
PGPR: plant growth promoting rhizobacteria
QV: quality value of epigenetic modification prediction
SMRT: Single-Molecule Real-Time Sequencing
UCMB: Ukrainian Collection of Microorganisms, section Bacteria (DK Zabolotny Institute of Microbiology and Virology, Kyiv, Ukraine).
