# Supplementary material for: Genetic, Epigenetic and Phenotypic Diversity of Four Bacillus velezensis Strains Used for Plant Protection or as Probiotics
Source: Front Microbiol. 2019 Nov 15;10:2610. doi: 10.3389/fmicb.2019.02610 (PMC6873887; doi:10.3389/fmicb.2019.02610)
Supplement: Supplementary file 1 [file Table_1.DOCX]

**Supplementary Table 1 |** Disease progression indices in groups of tomato seedlings infected with *R. solanacearum* and treated with selected *B. velezensis* strains or with water in the disease positive control. Each group comprised of fifteen plants. Plants were inspected weekly over 28 days after infection and treatment with *Bacillus* spores. Tomato wilting was scored according to the following disease index: 0 – no symptoms (healthy plant); 1 – one leaf partially wilted; 2 – two-three wilted leaves; 3 – all but 1 to 3 leaves are wilted; 4 – all leaves are wilted; 5 – plant died. Disease progression curves were plotted and Areas Under Disease Progression Curve (AUDPC) were calculated using the recorded indices. Smaller AUDCP indicates the better plant prevention.

| **Group** | **Day of inspection** | | | | | **AUDPC** | **AUDPC (%)** |
| --- | --- | --- | --- | --- | --- | --- | --- |
|  | 0 | 7 | 14 | 21 | 28 |  |  |
| UCMB5113 | 0 | 0 | 0 | 0 – 1 | 0 – 2 | 0 – 17 | 0 – 30% |
| UCMB5044 | 0 | 0 | 0 – 1 | 0 – 1 | 1 | 2 – 15 | 3 – 27% |
| UCMB5007 | 0 | 0 – 2 | 0 – 2 | 0 – 2 | 0 – 3 | 0 – 56 | 0 – 100% |
| Pos.Control | 0 | 0 – 1 | 0 – 2 | 0 – 2 | 0 – 3 | 0 – 49 | 0 – 88% |
| Max. Index | 0 | 2 | 2 | 2 | 3 | 56 | 100% |
