# Supplementary material for: Genetic, Epigenetic and Phenotypic Diversity of Four Bacillus velezensis Strains Used for Plant Protection or as Probiotics
Source: Front Microbiol. 2019 Nov 15;10:2610. doi: 10.3389/fmicb.2019.02610 (PMC6873887; doi:10.3389/fmicb.2019.02610)
Supplement: Supplementary file 3 [file Data_Sheet_2.PDF]

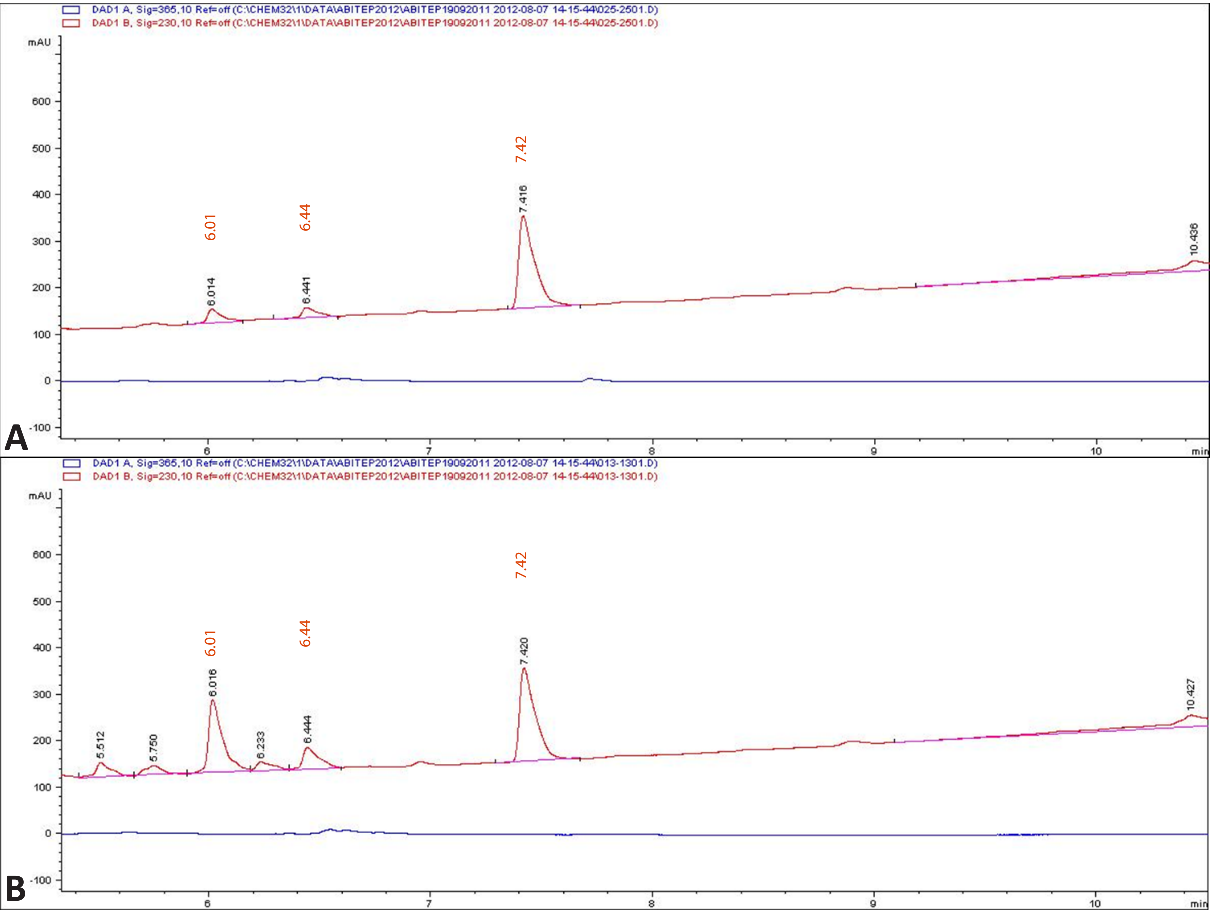

Supplementary Figure 2 | HPLC peaks for strain UCMB5044 A) 24 h cultivation; B) 48 h cultivation. OD values were measured at two wavelengths, 365 nm and 230 nm, depicted by blue and red lines, respectively. No characteristic peaks for known NRPS products were found.
