# Supplementary figures and images for: Genetic, Epigenetic and Phenotypic Diversity of Four Bacillus velezensis Strains Used for Plant Protection or as Probiotics
Source: Front Microbiol. 2019 Nov 15;10:2610. doi: 10.3389/fmicb.2019.02610 (PMC6873887; doi:10.3389/fmicb.2019.02610)

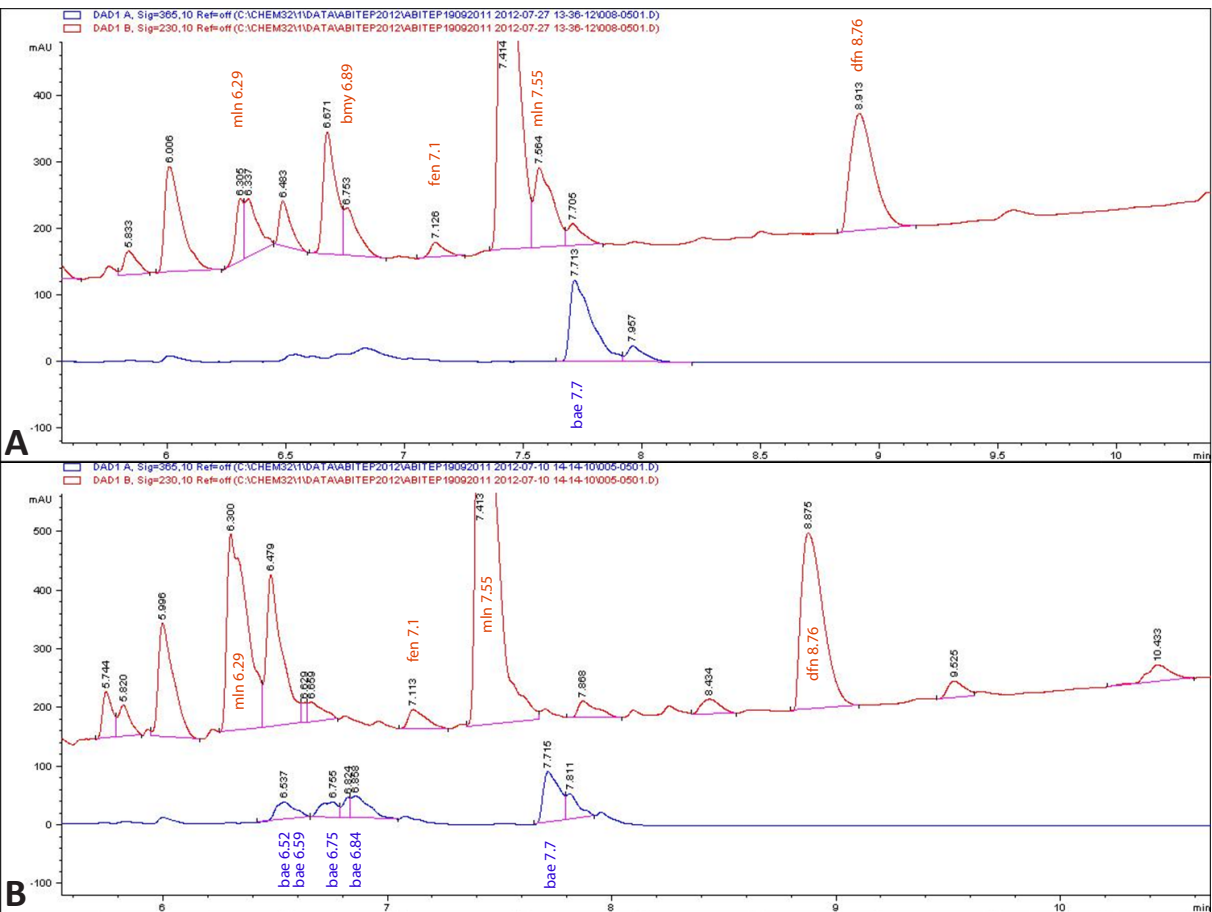

Supplement: Supplementary file 4 [file Data_Sheet_3.PDF]
