# Supplementary material for: Genetic, Epigenetic and Phenotypic Diversity of Four Bacillus velezensis Strains Used for Plant Protection or as Probiotics
Source: Front Microbiol. 2019 Nov 15;10:2610. doi: 10.3389/fmicb.2019.02610 (PMC6873887; doi:10.3389/fmicb.2019.02610)
Supplement: Supplementary file 6 [file Data_Sheet_5.PDF]

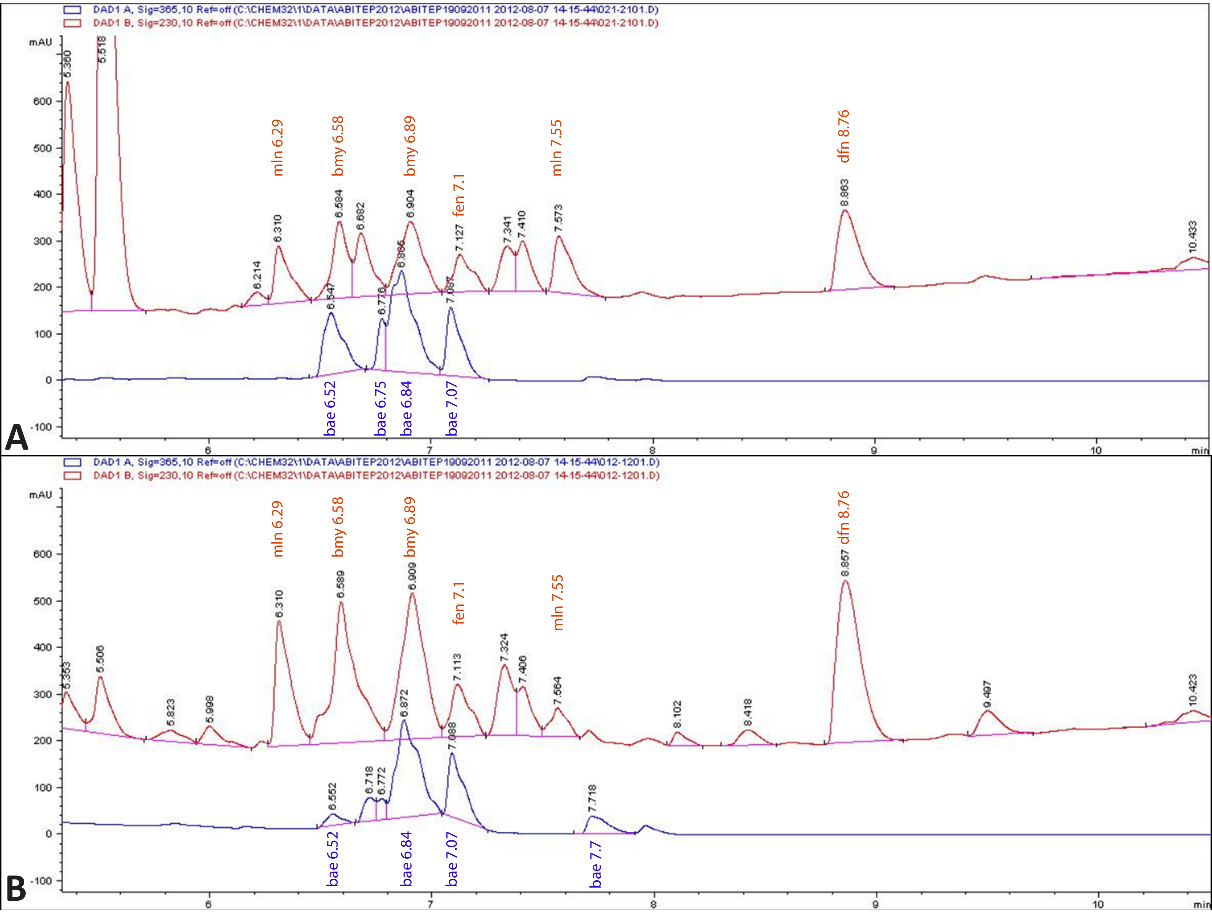

Supplementary Figure 5 | HPLC peaks for strain FZB42 A) 24 h cultivation; B) 48 h cultivation. OD values were measured at two wavelengths, 365 nm and 230 nm, depicted by blue and red lines, respectively. Characteristic peaks and the time of elution in seconds for bacillomycin (bmy), fengycin (fen), bacillaene (bae), difficidin (dif) and macrolactin (mln) are depicted.
