# Supplementary material for: Genetic, Epigenetic and Phenotypic Diversity of Four Bacillus velezensis Strains Used for Plant Protection or as Probiotics
Source: Front Microbiol. 2019 Nov 15;10:2610. doi: 10.3389/fmicb.2019.02610 (PMC6873887; doi:10.3389/fmicb.2019.02610)
Supplement: Supplementary file 7 [file Data_Sheet_6.PDF]

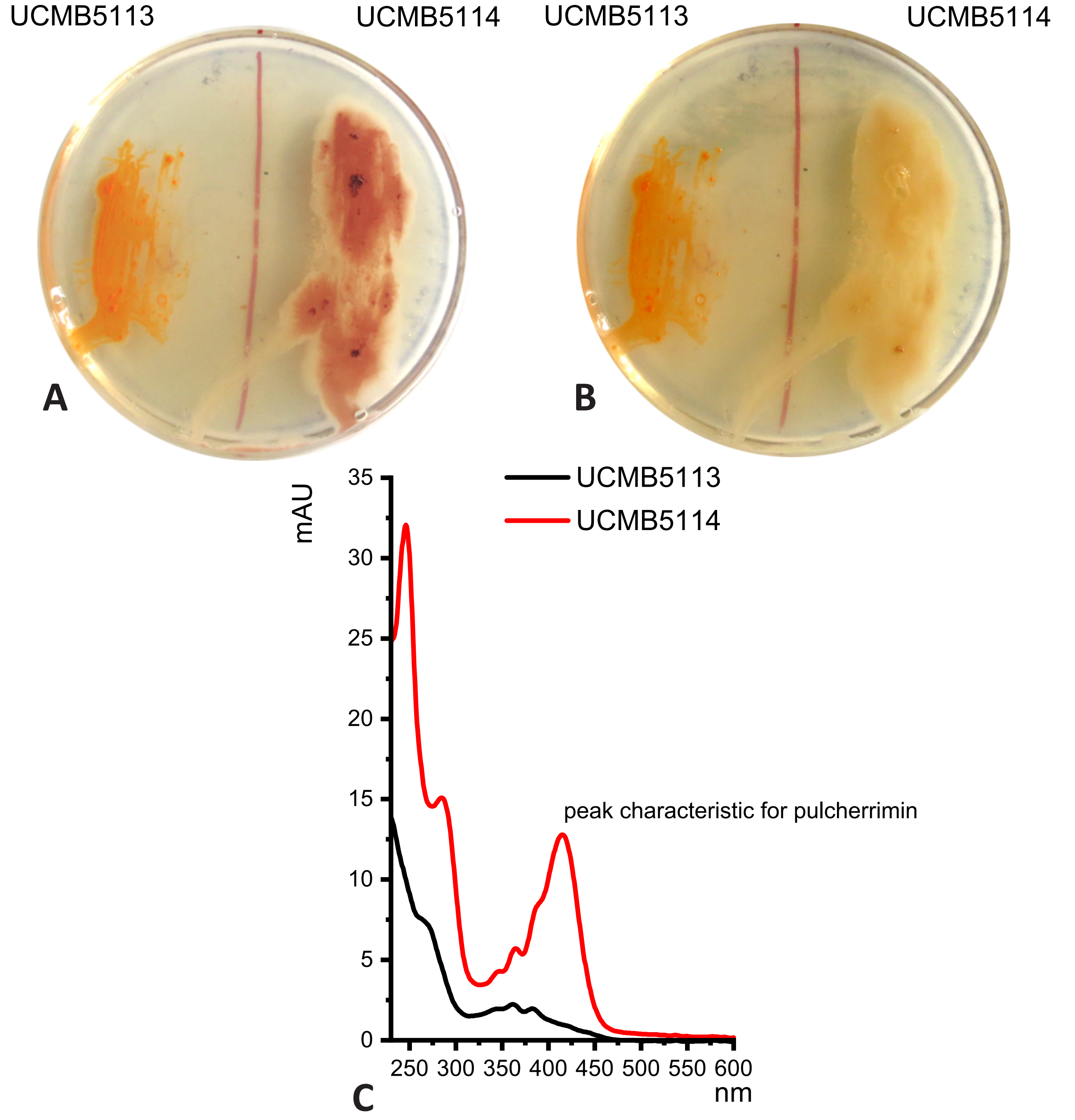

Supplementary Figure 6 | Pigmentation of the strain UCMB5113 in comparison to the pulcherrimin producing reference strain UCMB5114. A) Pigmented colonies of UCMB5113 and UCMB5114 on solid medium. B) The same colonies after the treatment with 1:1 methanol : 50% KOH. UCMB5114 changed the colour due to extraction and removal of pulcherrimin while the colour of the UCMB5113 colony remained the same.
C) UV-Vis spectra of the extracts from UCMB5113 and UCMB5114. A peak characteristic for pulcherrimin at 420-450 nm was observed only with the extract from the reference strain UCMB5114.
